# The Distinct Gene Regulatory Network of Myoglobin in Prostate and Breast Cancer

**DOI:** 10.1371/journal.pone.0142662

**Published:** 2015-11-11

**Authors:** Anne Bicker, Alexandra M. Brahmer, Sebastian Meller, Glen Kristiansen, Thomas A. Gorr, Thomas Hankeln

**Affiliations:** 1 Institute of Molecular Genetics, Johannes Gutenberg University, Mainz, Germany; 2 Institute of Pathology, University Hospital Bonn, Bonn, Germany; 3 Institute of Veterinary Physiology, Vetsuisse Faculty, University of Zurich, Zurich, Switzerland; 4 Regenerative Medicine Program, University and University Hospital Zurich, Zurich, Switzerland; Northern Institute for Cancer Research, UNITED KINGDOM

## Abstract

Myoglobin (MB) is not only strongly expressed in myocytes, but also at much lower levels in different cancer entities. 40% of breast tumors are MB-positive, with the globin being co-expressed with markers of tumor hypoxia in a proportion of cases. In breast cancer, MB expression is associated with a positive hormone receptor status and patient prognosis. In prostate cancer, another hormone-dependent cancer type, 53% of tumors were recently shown to express MB. Especially in more aggressive prostate cancer specimen MB expression also correlates with increased patient survival rates. Both findings might be due to tumor-suppressing properties of MB in cancer cells. In contrast to muscle, *MB* transcription in breast and prostate cancer mainly depends on a novel, alternative promoter site. We show here that its associated transcripts can be upregulated by hypoxia and downregulated by estrogens and androgens in MCF7 breast and LNCaP prostate cancer cells, respectively. Bioinformatic data mining of epigenetic histone marks and experimental verification reveal a hitherto uncharacterized transcriptional network that drives the regulation of the *MB* gene in cancer cells. We identified candidate hormone-receptor binding elements that may interact with the cancer-associated *MB* promoter to decrease its activity in breast and prostate cancer cells. Additionally, a regulatory element, 250 kb downstream of the promoter, acts as a hypoxia-inducible site within the transcriptional machinery. Understanding the distinct regulation of *MB* in tumors will improve unraveling the respiratory protein’s function in the cancer context and may provide new starting points for developing therapeutic strategies.

## Introduction

The respiratory protein myoglobin (MB) is expressed at high concentrations (~200–300 μM) in human skeletal and cardiac myocytes and, at much lower levels, in smooth muscle cells [1–3]. At its prosthetic heme group, the protein is able to bind gaseous ligands, with its main task being the transport and storage of O_2_ in myocytes [2,4]. Analyzing diving capacities of deep-diving mammals even revealed a direct correlation between myocytic Mb levels and O_2_ supply [5]. Although Mb -/- knockout mice are viable, they adapt to the loss of this heme protein by compensatory mechanisms, such as an increased capillary density and reduced cardiomyocyte width, to overcome the shortage of O_2_ supply [6,7]. In addition to its O_2_ storage/transport function in the cytoplasm, MB plays a crucial role in the detoxification of reactive oxygen and nitrogen species, i.e. by converting harmful excess NO to nitrate through the NO-dioxygenase activity of oxy-MB [8–10]. Under hypoxic conditions, deoxy-MB is instead able to produce NO, which in turn can inhibit mitochondrial O_2_ consumption and facilitate vasodilation in smooth muscle cells [11].

Besides its appearance in myocytes and few other somatic cell types, endogenous expression of MB has been reported for several tumor entities in humans at comparatively low micromolar levels, e.g. in breast cancer, non-small lung cancer, colon cancer, renal cell carcinoma, prostate cancer, osteosarcoma and leukemic bone marrow [12–18]. To investigate the biomedical relevance of MB expression in tumors, the survival rate of 917 primary breast cancer cases was monitored and correlated to their MB expression status. In a Kaplan-Meier analysis, patients with MB-positive breast tumors faced a beneficial prognostic outcome [15]. Kaplan-Meier plots of poorly differentiated prostate cancer entities (ranked Gleason 8–10) also showed a trend towards longer recurrence-free patient survival for MB-positive cases [18]. In contrast, high MB levels in lung adenocarcinoma correlated with a poor patient prognosis, although the study only compared the prognostic outcome of patients with high versus low MB-expressing tumors, but lacked the comparison to MB-negative tumor entities [16].

Up to now, little is known about the distinct function of MB in cancer cells. Signs of improved oxygenation were found in mice tumors generated by xenotransplantation of A549 lung cancer cells which ectopically expressed lentiviral-induced MB in the quantity range of skeletal muscle MB levels [19]. Similar to comparing the hearts of Mb -/- knockout versus wildtype mice, the MB-overexpressing xenotransplant tumors versus MB-negative controls showed altered vessel densities and reduced levels of the master transcription factor HIF1α, which accumulates in hypoxic/deoxygenated cells and tissues and triggers transcription activation of hypoxia-responsive downstream genes [19]. In addition, the MB-overexpressing tumors of xenotransplanted mice were more differentiated and had reduced local and distal metastatic spread [19]. In normal epithelial cancer cells, however, the overall MB level was roughly 300 fold lower than in the engineered xenografts [20]. Respirometry measurements in endogenously MB-expressing MDA-MB468 breast cancer cells even displayed lower cellular O_2_ uptake compared to *MB*-knockdown cells. Therefore the globin probably does not substantially contribute to O_2_ transport or storage in cancer cells [20]. MTT assays on MB-positive breast cancer cells indicated a decreased mitochondrial dehydrogenase activity compared to knockdowns under harsh hypoxia, suggesting that the antitumor effect of MB seen in cancer patients is exerted through mitochondria-impairing activities of the protein [20].

To pave the way for biomedical approaches exploiting such a tumor-suppressing property, recent studies addressed the mechanisms of *MB* gene regulation in cancer cells. Differing from the transcription machinery primarily active in myocytes, *MB* is mainly transcribed from a novel upstream promoter region in both, cancer cell lines and tumor biopsies [18,21]. A multitude of differentially spliced mRNAs possessing different 5’UTR exons but encoding the same protein sequence was recorded in tumor cells [21]. Amongst them, the mRNA variants 9, 10 and 11, all transcribed from the novel promoter upstream of exon 5u, were shown to be most prominently expressed [21]. Breast and prostate cancer biopsies revealed a significant correlation between increased levels of the alternative *MB* mRNA starting from exon 5u and the expression of hypoxia-responsive genes [18,21]. Accordingly, a hypoxic induction of *MB* mRNA was observed in breast and colon cancer cell lines cultivated under 1% O_2_ [21]. In part, this effect could be attributed to the transcriptional stimulation of *MB* expression by HIF1α and HIF2α transcription factors [20]. A hypoxia-responsive element (HRE) containing HIF1α binding sites was identified in an intronic region of the *MB* gene, which increased reporter gene expression by 43% in MDA-MB468 cancer cells upon experimental hypoxia [20]. Since total hypoxic induction of *MB* in the same cell line amounted to 3.5 fold, additional enhancer sites should be involved in *MB* mRNA upregulation in cancer cells. While investigating MB protein expression in prostate cancer, a significant positive correlation to androgen-receptor (AR) staining was observed and the analysis of publicly available transcriptome data suggested a dihydrotestosterone (DHT)-mediated suppression of the *MB* gene [18]. Among invasive breast cancer tumors, MB showed predominant expression in estrogen hormone receptor-positive, luminal-type breast tumor entities [15]. Estrogen treatment of MCF7 breast cancer cells resulted in a downregulation of *MB* mRNA expression, although the studies did not specify which *MB* variants were affected [14,15].

Thus, gene regulation of *MB* in the tumor context is complex and far from being understood in detail. To decipher *MB*’s regulatory components, we applied bioinformatic analyses of high-throughput sequencing data and identified novel potential enhancer regions that may interact with the *MB* promoter and participate in the regulatory network that triggers *MB* expression in tumors. Moreover, we investigated the influence of hypoxia and hormones on *MB* transcription, linking these stimuli to MB-associated patient prognoses.

## Materials and Methods

### Bioinformatic analyses of *MB* expression in differently treated cancer cells

To investigate the influence of estrogens, androgens and hypoxia on the splice variant-specific *MB* expression in MCF7 and LNCaP cells, RNA-Seq transcriptome datasets (see S1 Table for accession numbers) were downloaded from the NCBI sequence read archive (SRA) and processed with the CLC Genomics Workbench 6.5.1. (CLC Bio, Qiagen). Illumina adapters and low quality sequences (below 0.05) were filtered and 12 bp were trimmed from the 5’end and 5 bp at the 3’end. The minimum sequence length was set to 15 bp, allowing up to two ambiguous nucleotides. Processed reads were then mapped against the annotated *Homo sapiens* genome (hg19, GRCh37), using the CLC Genomics Workbench 5.6.1 RNA-Seq tool at default settings with a maximum of 2 mismatches. Reads that mapped to the coordinates of either one of the *MB* gene’s (NG_007075.1) alternative start exons (1u, 2u, 4u, 5u, 8u, 9u, 10u) were counted and normalized on the according exon length and dataset size, generating RPKM values (here: reads per exon size in kb per 1 mio. mapped reads).

GRO-Seq datasets downloaded from the NCBI-SRA (accession numbers in S1 Table) were processed with the CLC Genomics Workbench 6.0.4. (CLC Bio, Qiagen) Illumina adapters and 12 bp at each read’s 5’end were trimmed with a minimum remaining sequence length of 10 bp. The quality limit filter was set to 0.01 and up to two ambiguous nucleotides were allowed. Mapping (CLC Genomics Workbench 6.0.4 tool RNA-Seq) was performed at default parameters with 3 mismatches at maximum. Reads mapping within the region 100 bp upstream to 500 bp downstream of each transcript’s start site were counted and normalized relative to the fragment size (0.6 kb) and the size of the dataset to generate RPKMs.

For RNA-Seq and GRO-Seq data, boxplots and One Way ANOVA statistics (p<0.05) followed by Tukey-Kramer *post hoc* tests when appropriate were conducted with Excel 2010 (Microsoft). Asterisks indicate statistically significant differences comparing (i) differences between RPKMs of control cell batches and treatment batches for each start site and (ii) differences between RPKMs of the standard MB start site 9u to all other start sites.

Genomic datasets of epigenetic DNA marks including ChIP-Seq, FAIRE-Seq and Bisulfite-Seq (see S2 Table for accession numbers) were downloaded from the NCBI-SRA, the NCBI-GEO, and the EMBL-EBIArrayExpress databases (http://www.ebi.ac.uk/arrayexpress). All datasets were processed with the Genomatix Mining Station (Genomatix Software). Prior to mapping, reads of a Phred score lower than 30 were rejected and 15 bp at the 5’end and 5 bp at the 3’end of each read were masked. Reads were then mapped to the *Homo sapiens* Genome (NCBI built 37) using the ElDorado tool version 08–2011 at a minimum match accuracy of 92%. For all datasets, the options *deep mapping* and *map with insertions/deletions* were chosen. Bisulfite-treated datasets were additionally mapped against accordingly modified genome versions.

For better dataset handling, only bisulfite mapping results on chromosome 22 were inspected and thus reduced unique mapping reads to those, which align to chromosome 22 by applying the Galaxy platform Filter tool 1.1.0 (http://galaxyproject.org). For visualization of methylated and unmethylated sites all datasets were converted into UCSC custom tracks by the Genomatix Genome Analyzer (GGA; Genomatix Software) Bed File Toolbox.

Mappings of ChIP-Seq datasets and according input-files were processed with the GGA ChIP-Seq workflow tool. Peak calling was done with the Genomatix NGS Analyzer tool at a window size of 50 bp and with the minimum number of reads per peak being automatically calculated by applying a Poisson distribution. For evaluation of clusters, EdgeR statistics were applied at (p<0.05); Threshold: log2(fold change) ≥ 1 for enrichment and log2(fold change) ≤-1 for depletion. The generated ChIP-peak coordinates and the FAIRE-Seq mappings were again reformatted to UCSC custom tracks by the GGA Bed File Toolbox.

To gain more comprehensive information about the epigenetic profile of *MB* expressing cancer cell lines, additional mapping files were downloaded (S2 Table), including those generated by DNAse I HS-Seq and ChIA-PET and additional FAIRE-Seq and ChIP-Seq files [sources: The ChIA-PET Browser (http://chiapet.gis.a-star.edu.sg/); NCBI-GEO; EMBL-EBIArrayExpress database and the Database of Transcriptional Start Sites (http://dbtss.hgc.jp)]. Peak calling of DNAse I HS-Seq mappings was accomplished as described above for ChIP-Seq outputs. All datasets were transformed into UCSC custom tracks. Site-specific comparison and interpretation of generated custom tracks was performed in the UCSC genome browser (https://genome.ucsc.edu).

### Cell culture conditions

The breast cancer cell line MCF7 (gift by Prof. Dr. Pflugfelder, originally from ATCC; ATCC-HTB22) and the prostate cancer cell line LNCaP (gift by Dr. Szafranski, originally from DSMZ; ACC256) were cultured with phenol red-free DMEM (PAN P04-01158) and phenol red-free RPMI1640 (PAN P04-16516), respectively, both supplemented with stable glutamine (PAN), 10% FBS Gold (PAA) and 1% Pen/Strep (PAA). Incubation took place in an IG150 incubator (Jouan) at 37°C and 5% CO_2_ in a H_2_O-saturated atmosphere for normoxic culture conditions, while experimental hypoxia was provided in a CB 53 incubator (Binder) at 37°C, 1% O_2_ and 5% CO_2_ in a H_2_O-saturated atmosphere. Approval of Mycoplasm negativity was conducted with the VenorGeM Mycoplasma PCR detection kit (Minerva Biolabs). Both cell lines were authenticated by their SNP profiles (Multiplexion).

### RNA preparation and quantitative real-time reverse-transcriptase PCR (qRT-PCR)

RNA from LNCaP cells raised under normoxia or at 1% O_2_ for 24 h was extracted with the RNeasy Mini Kit (Qiagen), including an on-column DNAse I digestion. RNA integrity was approved to be >9.2 by running on an Agilent 2100 Bioanalyzer. The superscript III RT kit (Life technologies) was used for cDNA synthesis from 900 ng of LNCaP RNA. *MB* variants were quantified by qRT-PCR with a standard curve approach as described in [21]. A primer list can be found in (S3 Table). For amplification, GoTaq qPCR Master Mix (Promega) was used in a total volume of 10 μl. Reactions were carried out in an ABI 7500 Fast Real Time PCR system (Applied Biosystems) at an annealing temperature of 57°C. Triplicate assays were measured in each run with a total of n = 3 biological replicates. For calibration, we quantified LMNB1 mRNA in each sample (primers enlisted in S3 Table) and thus normalized the quantity of each *MB* variant to the housekeeping gene. Boxplots were generated and One Way ANOVA statistics (p<0.05) with *post hoc* Tukey-Kramer tests were conducted with Excel 2010 (Microsoft). Asterisks show statistically significant differences (i) between normoxia cells and 1% O_2_ cells for each mRNA variant and (ii) between the standard *MB* transcript variant 2 and all other transcript variants in normoxic cells.

### Functional analysis of potential *MB* enhancers by dual luciferase reportergene assays (DLRA)

*In silico* investigations of epigenetic histone marks revealed nine DNA regions, which potentially trigger the transcriptional activity of the cancer active *MB* exon 5u promoter. These regions were amplified with the KAPAHiFi PCR Kit (Peqlab) on genomic DNA of MCF7 cells. At their 5’ends, forward and reverse PCR-primers were equipped with MluI and XhoI restriction enzyme recognition sites, respectively (S3 Table). For cloning purposes, PCR amplicons and luciferase reporter gene vectors (pGL3-promoter vectors, Promega) were digested with FastDigest MluI and FastDigest XhoI enzymes (Thermo Scientific). After dephosphorylation of the 5’ends of the vector by the FastAP Thermosensitive Alkaline Phosphatase (Thermo Scientific), T4 ligase (Thermo Scientific) was used to ligate the PCR products into pGL3 vectors. The samples were transformed into DH10B bacteria host cells, selected and raised for plasmid preparations with the PureYield Plasmid Miniprep System (Promega). Sanger-based sequencing (StarSEQ) approved the accuracy of the cloned inserts. Alongsite with the reportergene constructs of potential enhancers, a 2072 bp spanning PCR amplicon of the *MB* exon 5u promoter region (S3 Table) was cloned into the pGL3 control vector (Promega) as described in [21].

To investigate the impact of the cloned promoter and enhancer sites on the target genes’ transcriptional efficiencies, reporter gene assays were conducted. To this end, ~10^4^ LNCaP or MCF7 cells were seeded in white Polystyrene 96 well plates (Thermoscientific, 136101) and transfected with a mixture of 91 μg pGL3 firefly luciferase plasmid, 9 μg pGL4.74 renilla luciferase vector (Promega) as an internal control and 0.2 μl FuGeneHD transfection reagent (Promega). 44 h post transfection, DLRA measurements were carried out in triplicates in a Glomax 96 well luminometer (Promega). For each well, 40 μl of both DLRA-system reagents (Promega) were used. Firefly light units originating from pGL3 plasmids were normalized on renilla signals to calculate the relative light units (RLU) for each well. For each plate, samples were further normalized on their cloned insert size and the RLUs measured for the empty vector. RLUs of cells treated with 100 nM estrogen (E2) for 1 h or 100 nM androgen (DHT) for 3 h prior to luciferase assays were divided by the RLUs measured of control cells transfected with the equivalent plasmid but mock-treated with ethanol. Boxplots and two-sided Student’s T-tests to test for significance were calculated with Excel 2010 (Microsoft).

## Results

### Hypoxia increases *MB* expression in prostate and breast cancer cells

**Fig 1:**
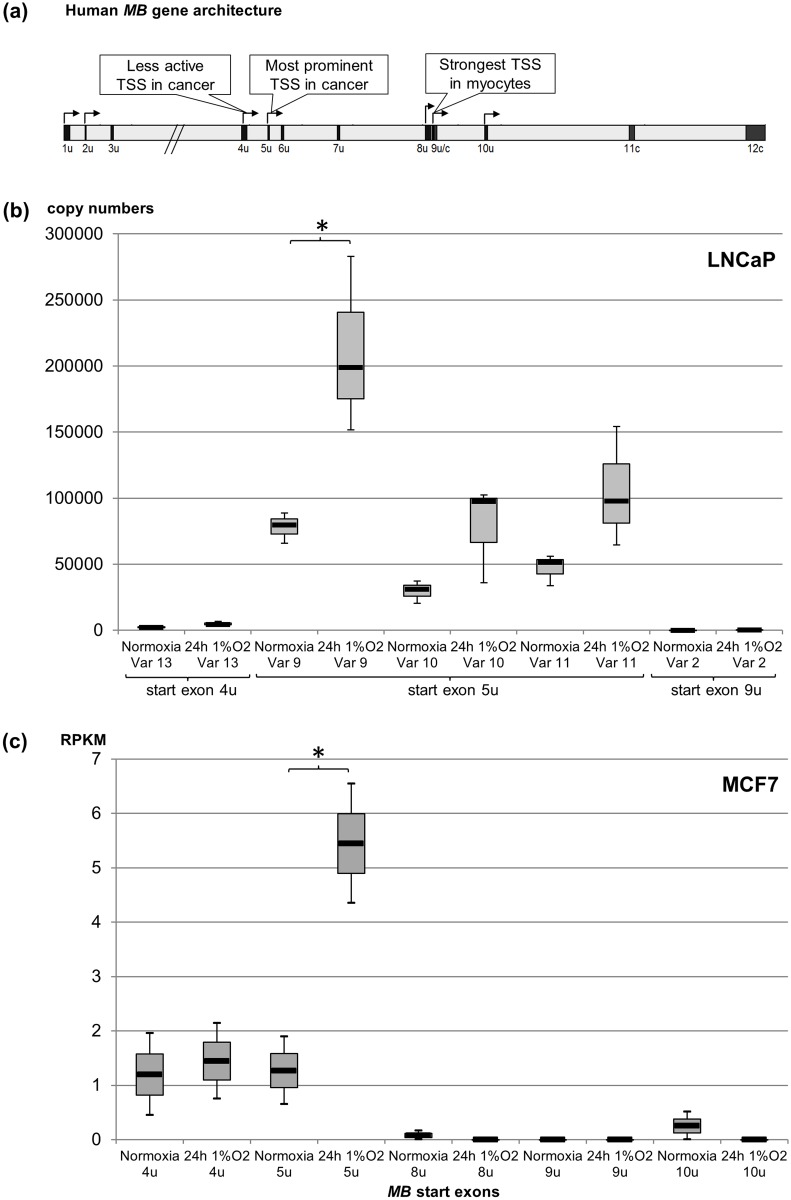
Expression analysis of *MB* transcript variants in normoxic and hypoxic (1%O_2,_ 24 h) cancer cells. **(a)** Architecture of the human *MB* gene. Boxes indicate exons, arrows mark transcription start sites (TSS) with the most active ones being labelled, based on [21]. **(b)** Quantification of cDNA copy numbers in LNCaP cells, measured in triplicate assays by transcript-specific qRT-PCR and normalized on LMNB1 controls (n = 3; * p < 0.05). mRNA copy numbers per 40 ng LNCaP total RNA are shown in box plots. **(c)** *In silico* quantification of *MB* transcripts by RNA-Seq analysis of MCF7 breast cancer cells (n = 2). Read counts are shown as RPKM values (* p < 0.05). Expression values are detailed in S1 Table.

In prostate cancer biopsies, mainly the alternative *MB* variants starting at upstream exon 5u were transcribed, while the *MB* transcript mainly expressed in myocytes (starting with exon 9u; Fig 1a) was hardly detectable [18]. To investigate if this regulatory pattern also applies to the prostate cancer cell line LNCaP, we performed splice variant-specific quantification of *MB* mRNAs by quantitative real-time reverse-transcriptase PCR (qRT-PCR). As in biopsies, LNCaP cells predominantly expressed transcripts starting with exon 5u. For comparison, *MB* mRNAs starting at exon 9u were expressed almost 2000 fold less than the variants starting at exon 5u (Fig 1b).

Previously, a 1.5 to 3.5 fold hypoxia-driven upregulation of the *MB* exon 5u mRNAs has been reported in two epithelial cancer cell lines of breast (MDA-MB468) and colon (DLD-1) origin [21]. In line with this, qRT-PCRs revealed on average a moderate 2.8 fold hypoxic induction of *MB* exon 5u transcripts in LNCaP cells (Fig 1b). To further monitor the effect of hypoxia on *MB* regulation in a second breast cancer cell line, MCF7, RNA-Seq data obtained from cells subjected to 1% O_2_ and normoxia for 24 h were downloaded (S1 Table) and mapped against the human reference genome. According to normalized read counts, hypoxic MCF7 cells expressed about 4.3 fold the amount of exon 5u *MB* transcripts than normoxic cells (Fig 1c). Hypoxia inducibility of *MB* mRNAs starting with exon 4u was 1.2 fold, with a relative exon 4u mRNA abundance of only 26% compared to the exon 5u transcripts under hypoxia. The exon 9u *MB* transcripts, most prominently expressed in myocytes, were not detected in MCF7 (Fig 1c, S1 Table). According tendencies were observed in an additional study (S1a Fig). Altogether, these expression data justify broad usage of the LNCaP and MCF7 cell models to study the *MB* gene regulatory network of *MB* primarily active in cancer cells.

### Downregulation of *MB* in prostate and breast cancer cell lines upon hormone treatment

**Fig 2:**
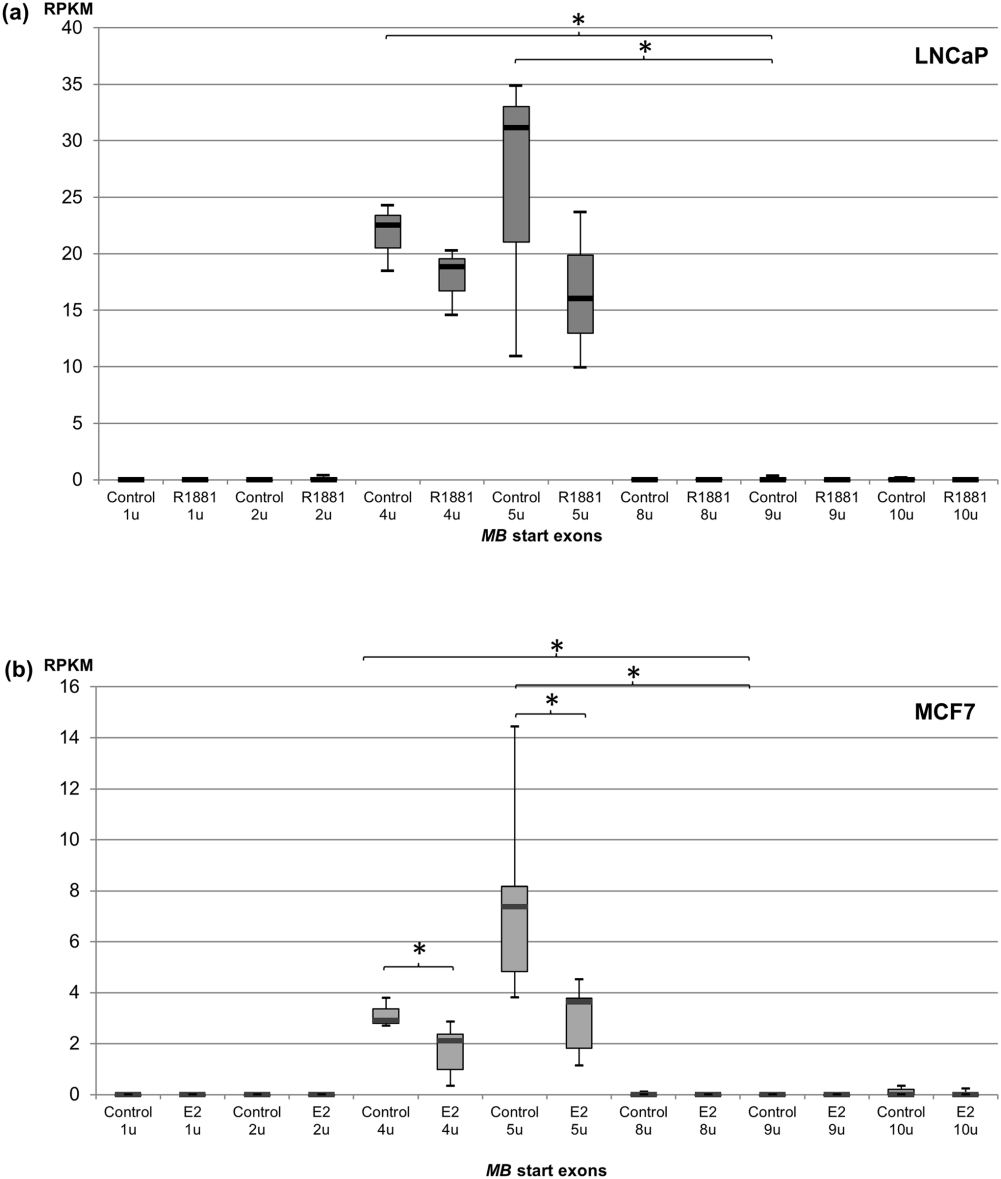
RNA-Seq based quantification of *MB* transcript variants in hormone treated cancer cells. **(a)** Box plots of *MB* variant expression in androgen (R1881) treated LNCaP cells after 12h versus non-treated controls (n = 3; * p < 0.05). Average transcript-specific expression values are listed in S1 Table. **(b)** *MB* variant expression in estrogen (E2) treated MCF7 cells after 24 h versus non-treated controls (n = 7). RPKM values of RNA-Seq read counts are shown as box plots, representing transcriptional levels of the different *MB* start exons (* p < 0.05). Average transcript-specific expression values are given in S1 Table.

To investigate how hormone application affects the expression of *MB* in LNCaP prostate cancer cells, transcriptome datasets treated with the androgen methyltrienolone (R1881) and solvent-treated control cells were evaluated for transcript-specific *MB* expression by read mapping (S1 Table). The vast majority of *MB* mRNAs expressed in LNCaP cells started at exons 5u and 4u. In contrast, the *MB* mRNA types predominant in myocytes were detected at much lower levels (e.g. exon 9u transcripts were expressed 214 fold less than exon 5u mRNAs; Fig 2a, S1 Table). Compared with controls, expression of *MB* exon 5u mRNAs in LNCaP cells incubated with 1 nM R1881 for 12 h was decreased to 64%. Exon 4u mRNA levels also slightly decreased to 82% after R1881 treatment (Fig 2a). *In silico* analysis of an independent second RNA-Seq dataset of R1881-treated LNCaP cells revealed that the androgen-mediated decrease of *MB* mRNAs occurred in a time-dependent manner (S2 Fig). After 24 h and 48 h, exon 5u mRNA levels decreased to 32% and 16%, respectively, and exon 4u transcripts to 37% and 23%, relative to androgen-free controls (S2 Fig).

Initiated by the finding that *MB* transcription decreased in prostate cancer cells upon androgen treatment, we further investigated the potential effect of estrogen application on *MB* expression in the breast cancer cell line MCF7. To this end, RNA-Seq and Genomic Run-On sequencing (GRO-Seq) datasets of MCF7 cells either treated with 17β-estradiol (E2) or ethanol as control were downloaded and mapped against the human genome. Splice variant-specific read counting indicated that again the mRNAs starting at exon 5u were most abundantly expressed in the breast cancer cell line (Fig 2b). Compared to these exon 5u variants, about 60% fewer transcripts starting at exon 4u were counted, while the muscle-associated *MB* transcripts were hardly detectable in MCF7 (S1 Table). After 24 h of E2 treatment, *MB* exon 5u mRNA expression decreased to 40% compared to controls, while exon 4u transcripts were downregulated to 55% upon hormone administration (Fig 2b). As previously observed in LNCaP cells, GRO-Seq data showed that the estrogen-driven downregulation of exon 5u *MB* transcripts in MCF7 cells also occurred in a time-dependent manner (S1b Fig). Treatment with 100 nM E2 resulted in a decrease of exon 5u mRNAs to 70% after 10 min, to 47% after 25 min and to 24% after 40 min. For the exon 4u variant, a more moderate downregulation to 85% was monitored when comparing cells incubated with E2 for 40 min to the control cells (S1b Fig).

### Long-range interactions between distal DNA elements and the cancer-associated *MB* promoter

**Fig 3:**
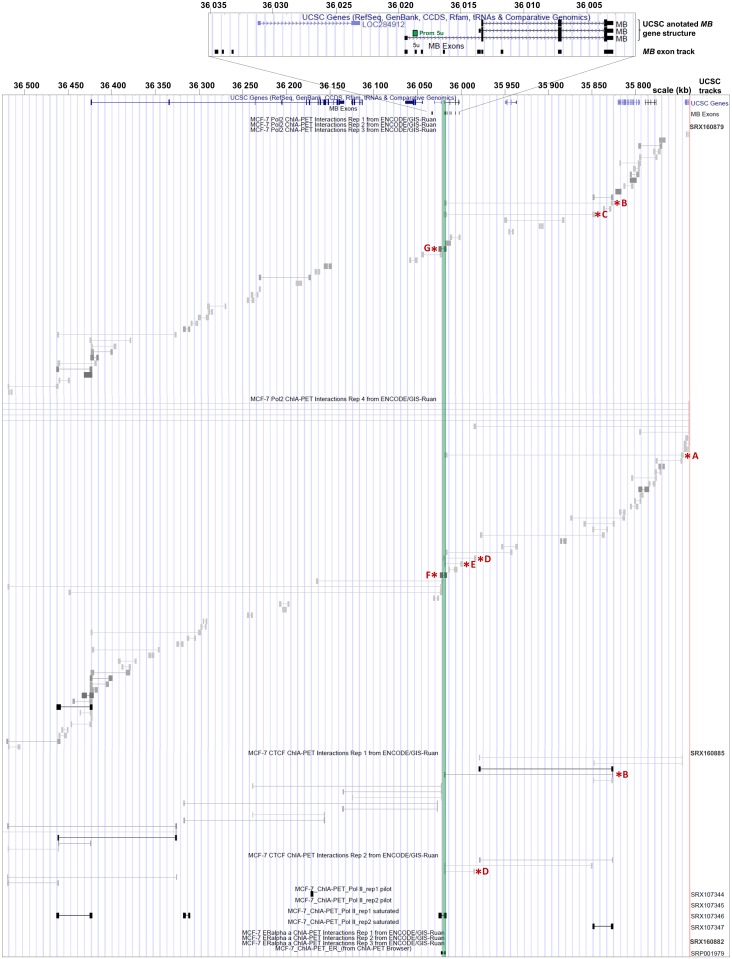
UCSC browser overview of ChIA-PET interactions around the 5u *MB* promoter in MCF7 cells. Top: UCSC annotation of *MB* and a custom track of all human *MB* exons, according to [21]. Bottom: UCSC browser and custom tracks with ChIA-PET coordinates. Dataset specifications are listed on the right. Asterisks indicate DNA regions that directly interact with the *MB* promoter. The green vertical line indicates the 5u *MB* promoter region.

Although the cancer-active *MB* promoter region was previously defined to be located in an interval 179 bp upstream to 25 bp downstream of exon 5u [21], little is yet known about its specific regulatory network. To this end, we studied publically available ChIA-PET (Chromatin interaction analysis by paired-end tag sequencing) datasets of MCF7 cells, which endogenously express *MB*. Each ChIA-PET marks two DNA regions that backloop in close proximity to each other, being specifically bound by proteins, i.e. transcription factors, which interact with each other [22]. Many of the RNA-Polymerase II-associated ChIA-PETs produced by the ENCODE consortium originate in the *MB* exon 5u promoter region and span to either one of five regions downstream (A-E) or one of two regions upstream (F, G) of the *MB* gene (Fig 3), encompassing a genomic region of 280 kb. Promoter interaction of three of these regions (B, C and G) was confirmed by RNA-PolII ChIA-PET tracks from independent datasets of the Gene Expression Omnibus (GEO) database (Fig 3). We further detected intrachromosomal inter-ligation ChIA-PETs, which reflect connections between enhancer regions, thus indicating the formation of a cloverleaf-like chromatin loop network around the *MB* exon 5u promoter.

In addition, ENCODE-UCSC tracks from CCCTC-binding factor (CTCF) ChIA-PET experiments indicated that regions B and D are bound by CTCF transcription factors while interacting with the *MB* exon 5u promoter via three-dimensional looping structures. DNA regions in close proximity to regions A and C are also bound by CTCF, possibly triggering the formation of a chromatin loop architecture (Fig 3). Region F also matches CTCF peaks, but the investigated ChIA-PET tracks lack evidence that this site directly interacts with the *MB* exon 5u promoter when bound to CTCF.

### Chromatin modifications at candidate *MB* enhancer sites

**Fig 4:**
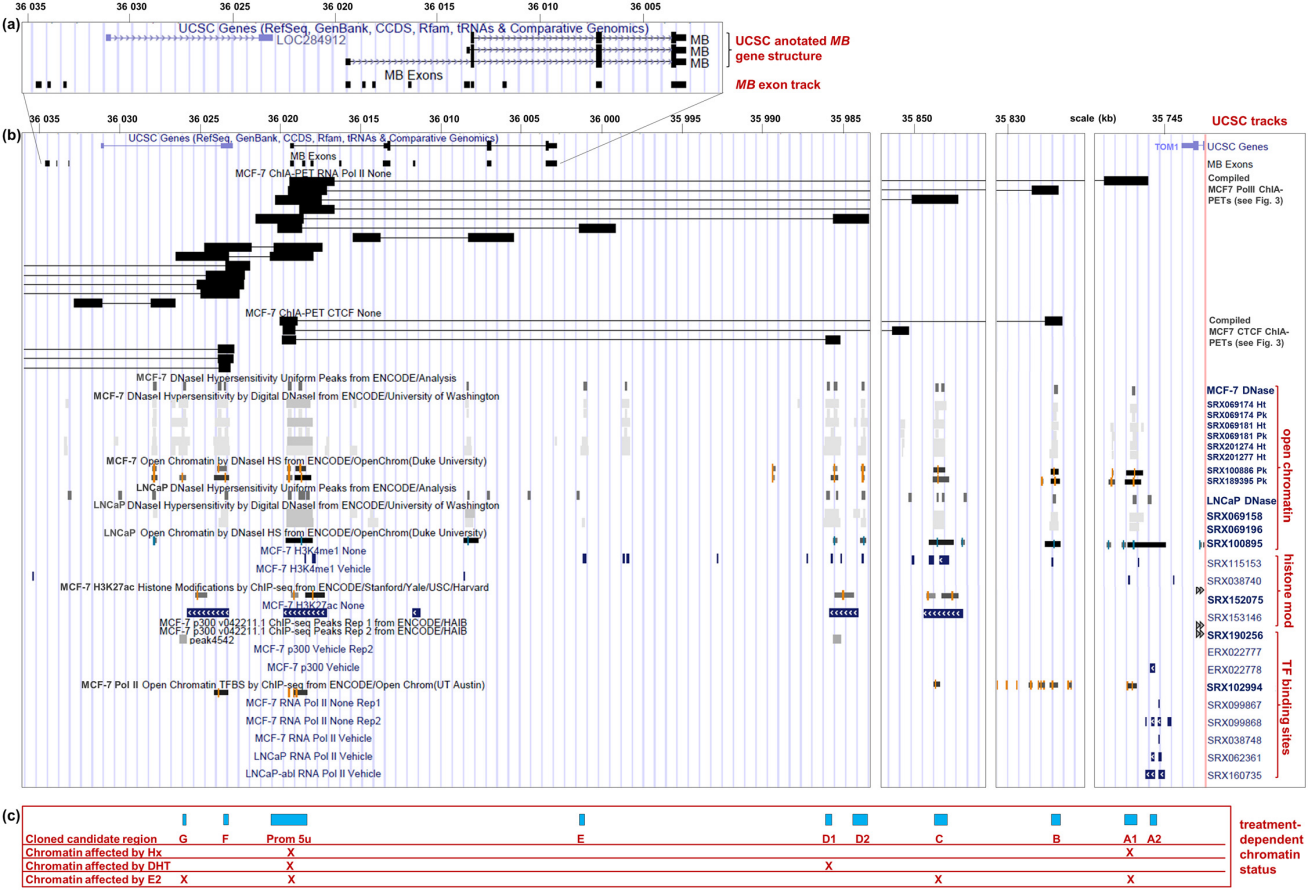
UCSC browser overview of ChIA-PET interactions and chromatin modifications around the *MB* exon 5u promoter. **(a)** UCSC annotation of *MB* and a custom track of all human *MB* exons, according to [21]. **(b)** UCSC browser and custom tracks with ChIA-PET coordinates. Chromosome 22 coordinates are written on top. The respective dataset specifications are listed on the right, grouped according to data showing open chromatin, histone modifications and transcription factor binding. Dark boxes in the upper section connected by thin lines reflect candidate interacting sequence regions according to ChIA-PET sequencing technique data mining. **(c)** Blue boxes indicate candidate enhancer regions, including the *MB* exon 5u promoter and DNA regions that directly interact with it based on ChIA-PET data and accumulative evidence from epigenetic marks as detailed above. The table further summarizes Fig 5a, Figures a in S3 and S4 Figs, crossmarking (X) which DNA sites show hypoxia-, androgen- and estrogen-associated chromatin modifications detected by ChIP-Seq and FAIRE-Seq.

To determine whether DNA regions A to G, which potentially interact with the *MB* exon 5u promoter based on ChIA-PET analyses, are indeed transcriptionally active, we investigated their chromatin status in more detail. Data mining of DNAse I hypersensitive site (DNAse I HS) peaks revealed that all of the sites hypothetically interacting with the promoter were indeed accessible for transcription factors in MCF7 cells, including the *MB* exon 5u promoter itself (Fig 4a and 4b). Additional DNAse I datasets from LNCaP cells also confirmed the promoter site and showed that all ChIA-PET matching DNA regions (despite G, where evidence was lacking) had an open chromatin status in prostate cancer cells. Analyzing chromatin immunoprecipitation sequencing (ChIP-Seq) datasets revealed a subset of histone marks (H3K4me1, H3K27ac) and basal components of the transcription machinery (e.g. PolII, p300) in each of the candidate regulatory DNA regions A1 to G, thus adding evidence to their involvement in transcriptional regulation (Fig 4b).

### Hypoxia-driven *MB* gene regulation by enhancer sites

**Fig 5:**
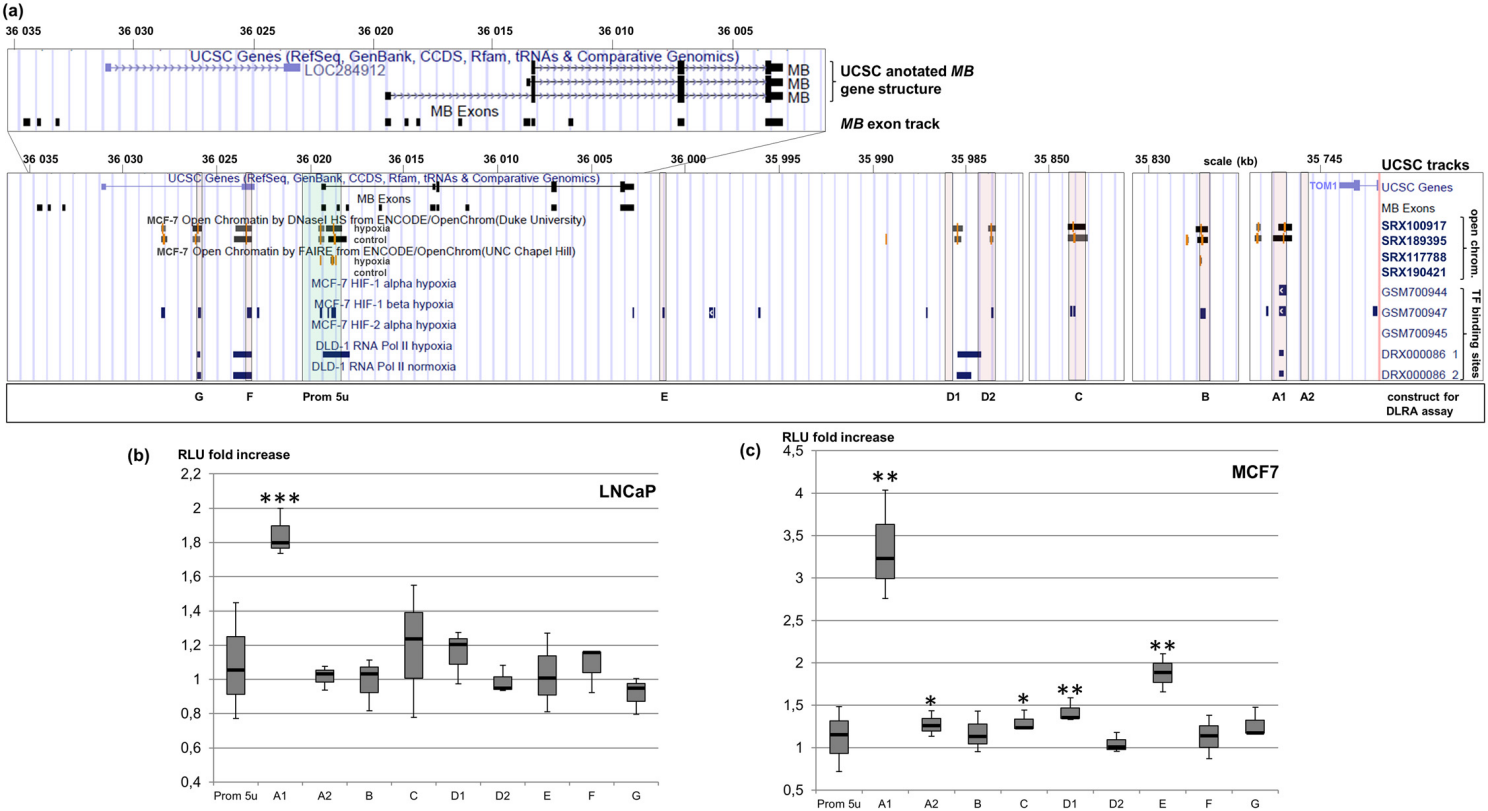
Identification of hypoxia associated regulatory regions of the *MB* gene. **(a)** UCSC browser overview of hypoxia associated chromatin modifications around the 5u *MB* promoter in MCF7 and DLD-1 cells. Top: UCSC annotation of the human *MB* gene and a custom track of all human *MB* exons, according to [21]. Bottom: UCSC browser and custom tracks. Dataset specifications are listed on the right. The green shattered box indicates the 5u *MB* promoter region. The red shattered boxes mark the DNA regions that directly interact with the 5u *MB* promoter site based on ChIA-PET data. The naming of the regions to match the DLRA measured constructs is written in the bottom line. **(b)** Hypoxia inducibility of the *MB* 5u promoter and interacting DNA regions in LNCaP cells. DLRAs were measured on hypoxia (1% O_2_ for 72 h) and normoxia incubated cells transfected with reportergene plasmids with different DNA regions. Box plots indicate the average RLU fold change of each construct under hypoxia versus normoxia growth conditions after normalization on empty vector constructs and renilla control vectors. Standard deviations are indicated by error bars (n = 3; *** p < 0.001). **(c)** Hypoxia inducibility of the *MB* 5u promoter and interacting DNA regions in MCF7 cells. DLRAs were measured on hypoxia (1% O_2_ for 72 h) and normoxia incubated cells transfected with reportergene plasmids with different DNA regions. Box plots indicate the average RLU fold change of each construct under hypoxia versus normoxia growth conditions after normalization on empty vector constructs and renilla control vectors. Standard deviations are indicated by error bars (n = 3; * p < 0.05; ** p < 0.01).

A possible activation of *MB* enhancer regions by hypoxia was investigated on the basis of DNAse I and ChIP-Seq datasets. No hypoxia-associated datasets of chromatin marks from LNCaP cells were available in public databases. However, DNAse I HS-Seq datasets of hypoxic MCF7 breast cancer cells and normoxic controls showed that eight out of nine ChIA-PET regions (except for E, where peaks were absent) and the *MB* exon 5u promoter region featured open chromatin not only under normoxia, but also under hypoxic culture conditions and thus were accessible for transcription factors. In DLD-1 colon cancer cells, which also endogenously express *MB* [21], RNA-PolII ChIP-Seq peaks confirmed the candidate enhancer regions A1, G and F to be active under both, hypoxia and normoxia (Fig 5a).

**Fig 6:**
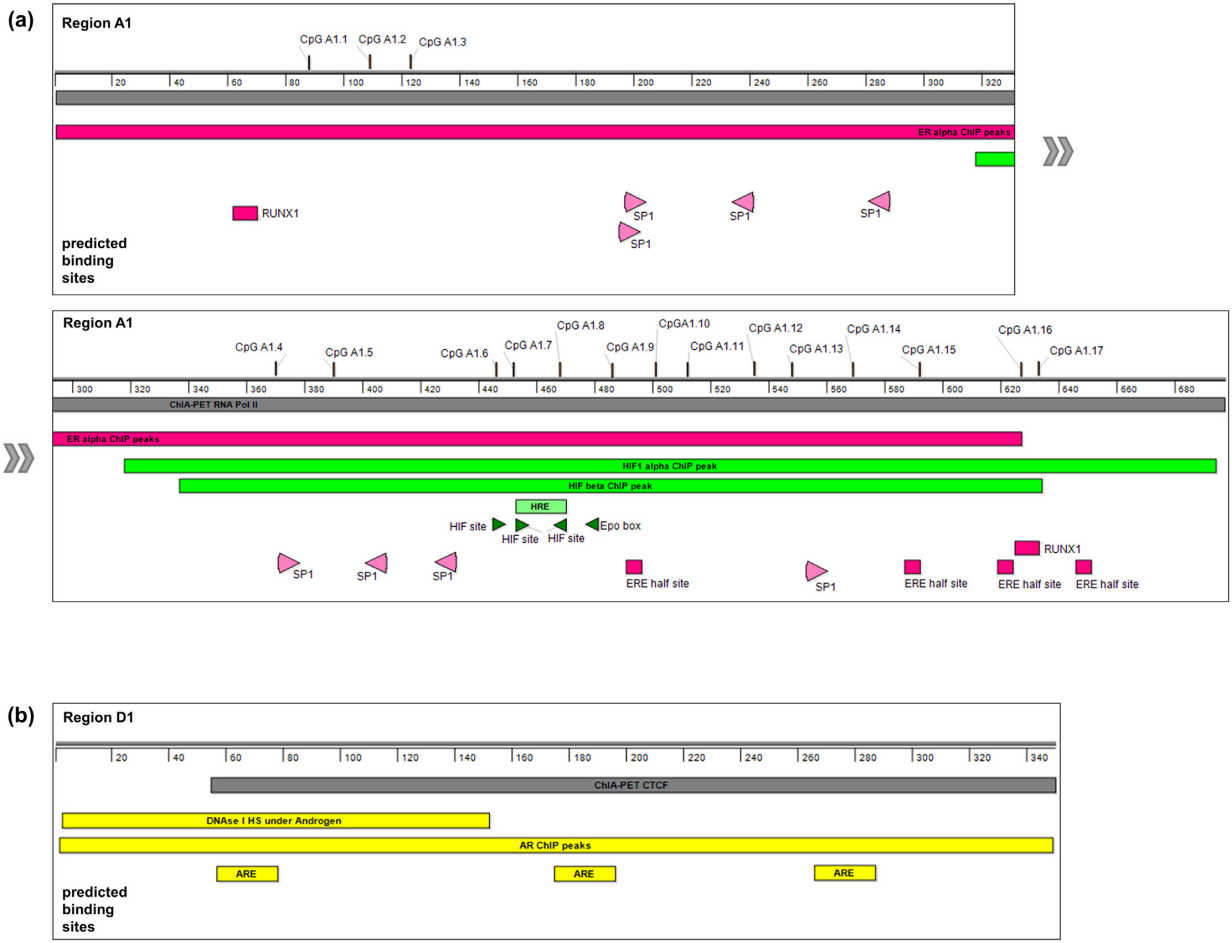
Evidence for potential functional sites in candidate *MB* regulatory regions. **(a)** Annotations of the hypoxia- and estrogen-responsive region A1. Large boxes indicate ChIP-Seq peaks and an RNA PolII-ChIA-PET. Small boxes and arrowheads show *in silico* predicted transcription factor binding motifs. CpGs whose methylation status has been revealed by bisulfite sequencing analyses are indicated (shown in detail in S4 Table). **(b)** Annotations of the androgen-responsive region D1. The large boxes indicate AR-ChIP-Seq peaks, a DNAse I hypersensitive sites and a CTCF-ChIA-PET. Small boxes show the locations of predicted transcription factor binding motifs (AREs).

ChIP-Seq experiments on MCF7 cells using either HIF1α or HIF1β antibodies indicated possible binding sites of the hypoxia-responsive master transcription factor HIF1 (Fig 5a). Our analyses revealed a HIF1α peak only for region A1, and this HIF1α peak is in close proximity to a HIF1β peak, whereby this region is most likely bound by the HIF dimer under hypoxia. Dual luciferase reportergene assays (DLRA) conducted in MCF7 and LNCaP cells with transfected plasmids containing either one of the potential enhancer regions A to G proved the hypoxia-responsiveness of region A1 in both cancer cell types. Compared to normoxia, relative light units increased 3.2 fold in MCF7 cells and 1.8 fold in LNCaP cells, when culturing each cell line transfected with A1 constructs under 1% O_2_ (Fig 5b and 5c). Noteworthy, this candidate enhancer site A1 is located about 250 kb downstream of the *MB* exon 5u promoter. Binding of the HIF dimer transcription factor to A1 is likely to occur at an *in silico* predicted HRE sequence motif in the A1 DNA region (Fig 6a).

Candidate enhancer region E also increased *MB* exon 5u promoter activity in MCF7 cells (1.9 fold) under hypoxia (Fig 5c). However, this enhancer effect was not detected by DLRAs in LNCaP cells. We also did not find DNAse I HS or formaldehyde assisted isolation of regulatory elements sequencing (FAIRE-Seq) evidence for altered chromatin accessibility in MCF7 cells at enhancer site E due to hypoxic stimuli. Moreover, we did not identify HRE binding sites in enhancer E, which is why we assume that region A1 is most likely to trigger the hypoxia-driven upregulation of the *MB* exon 5u promoter.

Analyzing bisulfite-sequencing datasets of MCF7, LNCaP and MDA-MB468 cells revealed a multitude of CpGs to be unmethylated in region A1, suggesting that specific CpG methylation might be an additional mode of transcription regulation of the *MB* gene (S4 Table). Although no information about the methylation status of the CpGs proximate to the HRE in region A1 was given for LNCaP cells, they were detected as unmethylated in both breast cancer cell lines, thus underlining the crucial role of this HRE in hypoxia-driven *MB* expression (S4 Table and Fig 6a).

### Hormone-driven chromatin modifications at *MB* enhancer sites

According to RNA-Seq data, treatment of LNCaP cells with androgens associated with a decrease of *MB* mRNA levels (Fig 2a). Thus, androgen treatment may impact on *MB* gene regulatory elements to cause a repression of transcriptional activity. DLRAs performed on LNCaP cells transfected with the *MB* exon 5u promoter constructs revealed that the promoter itself does not alter transcription efficiency upon DHT treatment (Figure b in S3 Fig). Therefore most likely, the effect of *MB* downregulation mediated by DHT occurs by interaction of the promoter with hormone-responsive enhancer sites. DLRAs revealed that none of the potential *MB*-regulating DNA regions studied as isolated DNA fragments enhanced activity in LNCaP cells if DHT-treated (Figure b in S3 Fig), but regions A1, B, C, D1, D2 and G slightly decreased reporter gene activity by 17 to 34% (Figure b in S3 Fig). Irrespective of androgen treatment, most regions that potentially loop back to the *MB* exon 5u promoter in LNCaP cells *in vivo* featured an open chromatin status (Figure a in S3 Fig). Part of region D1, however, only featured DNAse I peaks in the presence of androgens, highlighting this region as a candidate for hormone-responsiveness. Analysis of AR-ChIP-Seq data from LNCaP cells further revealed ChIP peaks in region D1 only present upon DHT treatment (Figure a in S3 Fig). Within this ChIP peak, region D1 contains three candidate androgen-responsive elements (ARE), identified by bioinformatic motif predictions (Fig 6b). This accumulation of evidence renders D1 a most promising candidate for androgen-mediated regulation in prostate cancer cells.

In a parallel analysis, we investigated hormone regulation of *MB* in MCF7 breast cancer cells, where estrogen triggered a decrease in *MB* mRNA levels (Fig 2b). Except for region A2, all candidate sites that interact with the *MB* promoter and the promoter itself revealed an open chromatin status both upon and without E2 treatment (Figure a in S4 Fig). Analysis of ChIP-Seq datasets using estrogen receptor α (ERα) antibodies on E2-treated MCF7 cells pointed out those DNA regions where transcription factors bind specifically upon addition of E2. In presence of the hormone, region A1 showed binding of the transcription factor complexes p300, RNA-Pol II and ERα (Figure a in S4 Fig). Thus, A1 is likely to play a crucial role in the E2-mediated regulation of *MB* transcription. In addition, we found ERα associated complexes to interact with DNA regions C and G upon E2 treatment, though the ChIP-peaks were only detected in one dataset each (Figure a in S4 Fig). Interaction of DNA region F with the *MB* exon 5u promoter under E2 treatment was indicated by a dataset of MCF7 cells from the ChIA-PET browser using ERα antibodies, although there were no additional ChIP-peaks evidencing the binding of ERα to DNA region F (Fig 3 and Figure a in S4 Fig). DLRA measurements in E2-treated MCF7 cells showed that neither the *MB* exon 5u promoter, nor any of the candidate interacting regions acted as transcriptional enhancers. Therefore, none of these DNA elements would counteract the experimentally determined E2-driven downregulation of *MB*. DLRA measurements further revealed that DNA region A1 significantly decreased transcriptional activity of the reporter gene construct by 27%, while the *MB* exon 5u promoter itself and DNA regions C and G showed a slightly reduced transcriptional activity of 16% and 7%, respectively (Figure b in S4 Fig). Four estrogen-responsive element (ERE) half sites and two runt-related transcription factor 1 (RUNX1) binding sites were detected bioinformatically in region A1 and possibly participate in the estrogen-driven downregulation of *MB* transcription in breast cancer cells.

## Discussion

Myoglobin (MB), present at high concentrations in striated skeletal and heart myocytes, has recently also been detected at lower expression levels in a variety of cancer specimen [23]. Concurrently, the architecture of the human *MB* gene has been revised by inclusion of several novel upstream exons, which produce alternatively spliced mRNAs originating at novel promoter regions [21]. While the classic *MB* promoter (starting at exon 9u) only drives the transcription of *MB* in muscle cells, *MB* transcription in epithelial cancer cell lines and tumor entities is predominantly regulated by a novel, more upstream promoter starting at exon 5u [21]. Since MB expression apparently correlates with a more benign breast and prostate tumor phenotype [15, 18] it will be important to understand its gene regulation in the context of cancer.

### Breast and prostate cancer cells predominantly express alternative *MB* transcripts

Our *in silico* and qRT-PCR studies showed a predominant expression of alternative, non-standard *MB* mRNAs in MCF7 breast cancer and LNCaP prostate cancer cells. The *MB* transcript variants driven by the exon 5u upstream promoter were most abundantly expressed in these cell lines. At a minor level, transcripts starting at exon 4u were also detected, while *MB* transcripts initiated at the myocyte-active gene promoter were only found in minute amounts. These results were in full agreement with previously published quantitative data on *MB* splice variant amounts in MDA-MB468 breast cancer, DLD-1 colon cancer cells [21] and hematopoietic stem/progenitor cells [24]. By mining of epigenetic data (e.g. by ENCODE), we obtained broad evidence for the dominant activity and accessibility of the *MB* exon 5u promoter in cancer cells, while the classic myocyte-type *MB* promoter approved silent (Fig 4b). The data revealed a complex network of candidate enhancer sites that potentially interact with the *MB* exon 5u promoter to regulate its activity in cancer cells with respect to hypoxia and hormone stimuli.

### *MB* upregulation under hypoxia

In myocytes, *MB* expression is not inducible by hypoxia alone, but requires additional stimuli such as exercise [25]. Accordingly, the classic *MB* promoter lacks candidate HRE motifs [26]. In contrast, hypoxia induction of MB protein and mRNA was previously reported in HBEC/3KT, MCF7, MDA-MB468, DLD-1, HTB182 and renal cell carcinoma cell lines [14,16,17,20,21]. We show here that expression of *MB* exon 5u transcripts increased 2.8 fold and 4.3 fold in LNCaP and MCF7 cells, respectively, after 24 h at 1% O_2_ (Fig 1b and 1c). Up to now, we have a rather vague understanding of the impact of hypoxia and the precise contribution of HIF-dependent and -independent cascades on the regulation of the *MB* gene (e.g. [20]). One candidate HRE, upregulating *MB* by 43% under hypoxia *in vitro*, was recently described downstream of *MB* exon 7u [20]. Our current bioinformatic analyses in MCF7 cells, however, did not corroborate a prominent role of the exon 7u-linked HRE in the hypoxia regulation of *MB*. We thus focused on the detection of other, novel enhancer sites that may facilitate—even via long-distance activation—the hypoxia inducibility of the *MB* gene.

**Fig 7:**
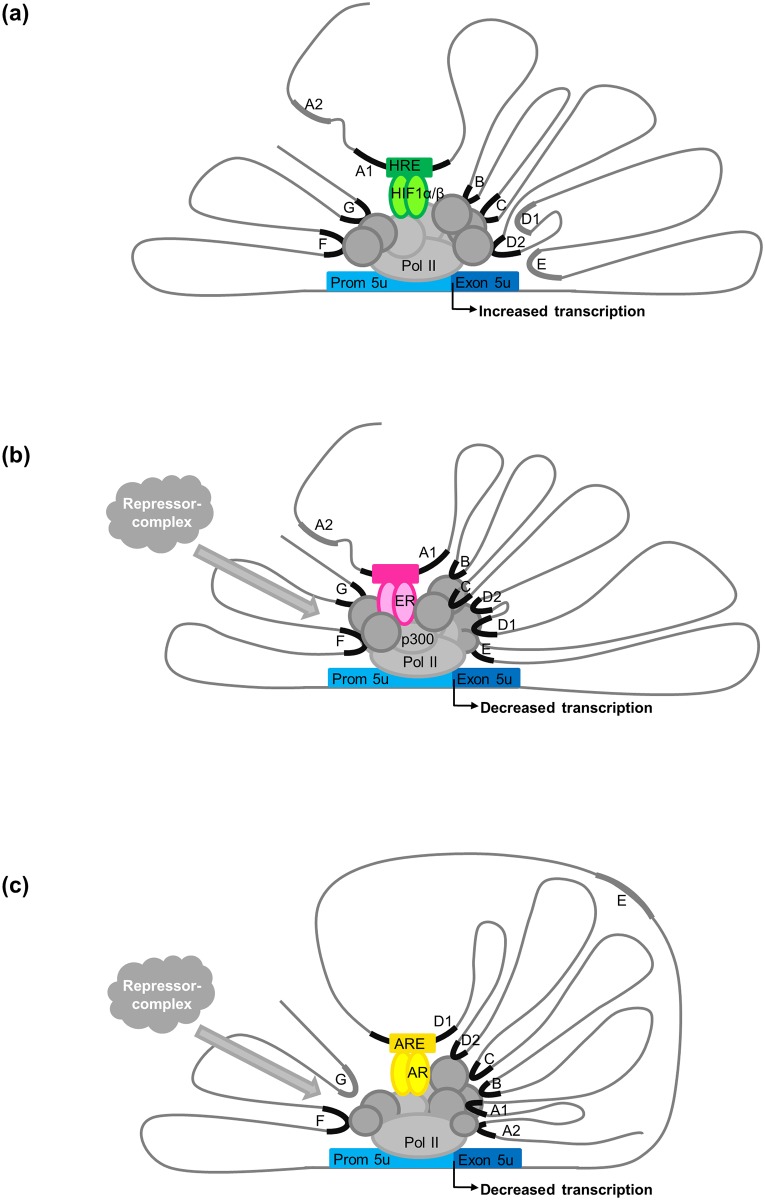
*MB* exon 5u promoter regulatory networks in tumor cells under different conditions. **(a)** Hypoxia (1% O_2_)-driven upregulation of the promoter in MCF7 cells. HIF1α/β binding to the candidate HRE in region A1 is indicated (Fig 6a). Regions G and F interact with RNA-PolII. Open chromatin regions are drawn in black (Fig 5a). **(b)** Estrogen (E2)-mediated downregulation of the promoter in MCF7 cells. ERα binding to region A1 may occur at the ERα-half sites and the RUNX1 site (see Fig 6a for details). The complex bound to region A1 further interacts with p300 and RNA-PolII. Regions C and G also interact with the ERα protein complex, region F associates with RNA-PolII (Figure a in S4 Fig). This interaction network recruits a repressor complex to downregulate the transcription of 5u *MB* transcripts under E2 treatment. Open chromatin regions are marked in black. **(c)** Androgen (DHT)-driven downregulation of the promoter in LNCaP cells. AR binding to a hypothetical ARE motif in region D1 (Fig 6b) is indicated. Region A2 interacts with RNA-PolII. This interaction network recruits a repressor complex to downregulate the transcription of exon 5u *MB* transcripts under DHT treatment. Open chromatin regions are marked in black (Figure a in S3 Fig).

In LNCaP and MCF7 cells, the *MB* exon 5u promoter itself did not account for hypoxia sensitivity, as shown by reporter gene assays (Fig 5b and 5c). Instead, our studies revealed a candidate enhancer site A1 in ~250kb distance to the promoter, which is hypoxia-responsive as confirmed by DLRA assays. Region A1 interacts with the promoter, as evidenced by PolII ChIA-PETs from MCF7 cells and features an open chromatin status according to FAIRE-Seq and DNAse I HS data (Figs 3 and 5a). The HIF1α ChIP-Seq peak in hypoxic MCF7 cells, which harbors a potential HRE sequence motif, lends additional evidence to a hypoxia responsive role of region A1. Bisulfite sequencing data further revealed the methylation status of four CpGs around the HRE (Fig 6a), which are all unmethylated in MCF7 and MDA-MB468, thus allowing for hypoxia-inducibility of the *MB* gene. In addition, several other DNA elements around the *MB* gene may form additional interactions with the A1 enhancer for full stimulation of the promoter (Fig 7a), as suggested by the open chromatin status of regions B, C, D2, F and G under normoxia and hypoxia according to FAIRE-Seq and DNAse I HS tracks (Fig 5a). The discovery of the HIF-bound HRE enhancer site provides a mechanistic basis for the finding that siRNA knockdown of HIF1α and HIF2α most efficiently counteracts induction of *MB* mRNA after 72 h under 1% O_2_ [20]. Importantly, *MB* upregulation by hypoxia does not only occur in various cell lines, but also in a broad range of epithelial cancer tumor entities [17,18,20,21]. However, the molecular function of hypoxia-upregulated, but still low-level expressed MB in tumor cells is currently unclear. As in smooth muscle cells [11], it is possible that deoxy-MB functions in hypoxic regions of epithelial cancers by producing NO, thereby activating NO signalling. Alternatively, MB may scavenge harmful ROS produced by intense fatty acid metabolism [27]. We further found a decreased mitochondrial dehydrogenase activity in MB-proficient MDA-MB468 breast cancer cells under stringent hypoxia (0.2% O_2_), compared to siRNA-mediated *MB* knockdown cells, suggesting that deoxy-MB is indeed able to exert unexpected respiration-delimiting functions in O_2_-deprived cancer cells [20]. Although the tumor suppressing effect of hypoxia-regulated MB expression in cancer cells thus possibly relies on the globin’s ability to either produce or scavenge NO/ROS, additional experiments are needed to specify MB’s suppressive role in tumors.

### Estrogen-mediated downregulation of *MB* expression in breast cancer cells

In addition to hypoxia regulation of *MB*, we previously discovered that exposure of ERα-positive [28] MCF7 breast cancer cells to 17β-estradiol (E2) resulted in a dose-dependent downregulation of *MB* [15]. Our results approved that expression of exon 5u *MB* transcripts, and also to a lesser extent of exon 4u mRNAs, significantly decreased upon hormone treatment in a time-dependent manner. Several lines of evidence support the hypothesis that the promoter itself is not hormone-responsive, but candidate enhancer sites are involved: (i) most candidate regions revealed open chromatin in both, E2-treated and -untreated MCF7 cells, (ii) ChIP-Seq data revealed binding of ERα after E2 treatment in DNA regions A1 (in four independent datasets), C and G, (iii) additional ChIP-Seq data supported p300 activator and RNA PolII binding in E2-treated cell batches only, and (iv) region A1 suppressed reportergene transcription in MCF7 cells after estrogen treatment (Fig 4c, S4 Fig). Transcription factor binding motif searches revealed a combination of SP1 boxes, RUNX1 motifs and ERE half sites in region A1, which are hypothetically involved in the hormone regulation of the *MB* exon 5u promoter. Based on ChIA-PET data mining, DNA regions A1 and C potentially associate in a cloverleaf-like loop architecture together with regions B and D1 to interact with the *MB* promoter under E2 treatment (Fig 7b). Three general mechanisms have been described for ERα/E2-mediated downregulation of target genes [29–31]. Of those, the “squelching model”, implying a displacement of transcription factor p300 from the repressed target gene [29], appears unlikely in the *MB* case, since we observed a p300 ChIP-Seq peak in region A1 after E2 treatment. Instead, transcriptional silencing of *MB* might have occurred via active recruitment of repressor-complex factors such as FOXA1 and HDAC7 [30] or by the repressor of estrogen receptor activity (REA) in combination with HDAC1 and HDAC2 [31].

In our working model, region A1 may represent a bi-functional gene regulatory element for *MB*, acting as an enhancer of *MB* transcription under hypoxia, and participating in downregulation upon estrogen exposure in an ER-positive background. Exon 5u *MB* mRNA expression in MCF7 cells was decreased by 30% already after 10 min and by 60% after 24 h upon E2 treatment (Fig 2b and S1b Fig). *MB* hypoxia regulation, in contrast, peaked after 72 h [20]. Thus, the hormone response appeared to occur much faster than the hypoxia response. We conclude that though *MB* expression might be downregulated by E2 treatment via enhancer A1 after a few minutes up to few hours, the strongest HIF1 response of *MB* was reported one to three days post hypoxia stimulus and thus must not necessarily interfere with the early E2 response.

In well-differentiated invasive ductal breast carcinomas, MB was described to be co-expressed with ERα and thus adds prognostic information to ERα-positive patient tumors [15,20]. Tumors of pre-menopausal patients revealed lower MB expression levels than those of post-menopausal patients [15]. We therefore expect the E2-mediated suppression of *MB* transcription in ERα positive breast tumors to be weakened, once E2 levels start to decrease in post-menopausal females. It is also not clear how the inherently more aggressive ER-negative and triple-negative (ER-, PR-, Her2-) breast cancer cells such as MDA-MB468 [28,32,33] will respond in *MB* expression when subjected to E2.

### Androgen downregulates *MB* in prostate cancer cells

Analogous to the E2 response in breast cancer, androgens were found to downregulate *MB* mRNA expression in prostate cancer cells. Our *in silico* analyses indicated that treatment of LNCaP cells with the potent, non-aromatizable R1881 androgen tended to decrease *MB* exon 5u transcripts to 64%, 32% and 16% of control levels after 12, 24 and 48 h of hormone exposure, respectively (Fig 2a and S2 Fig). This result is in line with a significant decrease of overall *MB* transcription described in LNCaP cells subjected to DHT for 16 h [18].

To unveil the regulatory network orchestrating the decrease of *MB* expression upon androgen treatment, we studied the enhancer activity of DNA regions which interact with the *MB* exon 5u promoter according to ChIA-PET data. Similar to the estradiol-mediated gene regulation, reporter gene assays revealed that the *MB* exon 5u promoter did not alter transcriptional activity in DHT-treated LNCaP cells. In contrast, a subset of candidate enhancer regions, which interact with the *MB* exon 5u promoter, slightly decreased transcription *in vitro*. Mining of three prostate cancer cell ChIP-Seq datasets revealed region D1, encoding an ARE sequence motif, to be bound by AR specifically upon DHT treatment. Moreover, DNAse I datasets proved accessibility of D1 for transcription factors in androgen-supplemented LNCaP cells. The looping chromatin structure of regions A1, B, C and D1 that we hypothesize to be formed in breast cancer cells to drive the E2-stimulated transcription repression of *MB* could also be formed in LNCaP cells in response to DHT treatment (Fig 7c). Like in the ERα scenario, the chromatin interaction network in LNCaP cells is likely to recruit AR repressor complex proteins to the promoter (Fig 7b) to mediate transcriptional repression of *MB* under androgen treatment [34,35].

In prostate cancer biopsies, immunohistochemistry staining revealed a co-localization of MB with AR and FOXA1 [18]. According to *in silico* data, LNCaP cells express elevated levels of *MB* after 3 weeks of androgen deprivation, compared with hormone-naive control cells treated with androgens [18]. A therapeutical approach to combat hormone-naive prostate cancer is to treat patients with androgen antagonists, thus preventing the AR from activating genes associated with tumor growth and survival. Based on our results, we assume that this therapy would prevent an AR-mediated decrease of *MB* transcripts, thereby indirectly ensuring higher levels of *MB* expression in prostate tumor cells. Increased *MB* expression, being associated with an ameliorated patient prognosis [18], could hypothetically contribute to the successful outcome of anti-androgen tumor therapies.

In conclusion, the availability and detailed analysis of publically accessible high-throughput epigenomic datasets present a formidable opportunity to dissect gene regulatory pathways of biomedically important genes and to infer important working hypotheses for functional studies.

## Supporting Information

S1 Fig Expression analysis of *MB* transcript variants in MCF7 cells. **(a)** *In silico* quantification of *MB* transcripts by RNA-Seq analysis in normoxic and hypoxic (1% O_2_, 24 h) cancer cells. Read counts are shown as RPKM values. Expression values are detailed in S1 Table. **(b)** Start-site specific *MB* expression in MCF7 cells treated with 100 mM E2 for different time periods. GRO-Seq reads which mapped 100 bp upstream to 500 bp downstream to each start site were counted and normalized to the fragments’ size and total reads of the dataset (in Mio). Box plots of (n = 2–3) datasets for each time period represent the transcriptional levels of *MB* start exons (* p < 0.05). Average transcript-specific expression values are given in S1 Table.
(TIF)

S2 Fig Expression of *MB* transcript variants in LNCaP cells treated with 1 nM R1881 for different time periods. *MB* start exon quantifications are shown as RPKM values for each experiment, indicating the expression of according mRNA variants. Expression values are detailed in S1 Table.
(TIF)

S3 Fig Identification of androgen associated regulatory regions of the *MB* gene in LNCaP cells. **(a)** UCSC browser overview of androgen associated chromatin modifications around the 5u *MB* promoter in LNCaP cells. Top: UCSC annotation of *MB* and a custom track of all human *MB* exons, according to [21]. Bottom: UCSC browser and custom tracks. Dataset specifications are listed on the right. The green shattered box indicates the 5u *MB* promoter region. The red shattered boxes mark the DNA regions that directly interact with the 5u *MB* promoter based on ChIA-PET data. The naming of the regions to match the DLRA measured constructs is written in the bottom line. **(b)** Dihydrotestosterone inducibility of the *MB* 5u promoter and interacting DNA regions in LNCaP cells. DLRAs were measured on 100 nM DHT treated (for 3 h) and control cells transfected with reportergene plasmids with different DNA regions. Box plots indicate the average RLU fold change of each construct measured in hormone treated versus control cells after normalization on empty vector constructs and renilla control vectors. Standard deviations are indicated by error bars (* p < 0.05; ** p < 0.01; n = 5).
(PDF)

S4 Fig Identification of estrogen associated regulatory regions of the *MB* gene in MCF7 cells. **(a)** UCSC browser overview of estrogen associated chromatin modifications around the 5u *MB* promoter in MCF7 cells. Top: UCSC annotation of the human *MB* gene and a custom track of all human *MB* exons, according to [21]. Bottom: UCSC browser and custom tracks. Dataset specifications are listed on the right. The green shattered box indicates the 5u *MB* promoter region. The red shattered boxes mark the DNA regions that directly interact with the 5u *MB* promoter based on ChIA-PET data. The naming of the regions to match the DLRA measured constructs is written in the bottom line. **(b)** Estrogen inducibility of the *MB* 5u promoter and interacting DNA regions in MCF7 cells. DLRAs were conducted on 100 nM E2 treated (for 1 h) and control cells transfected with reportergene plasmids with different DNA regions. Box plots indicate the average RLU fold change of each construct measured in hormone treated versus control cells after normalization on empty vector constructs and renilla control vectors. Standard deviations are indicated by error bars (* p < 0.05; ** p < 0.01; *** p < 0.001; n = 3).
(PDF)

S1 Table RNA-Seq and GRO-Seq datasets analyzed from the NCBI-Sequence Read Archive. RNA-Seq reads mapping to each *MB* start exon were counted and normalized as RPKM values in order to estimate the average start-site specific expression of different *MB* variants. Detailed box plots of all studies are shown in Figs 1c, 2a and 2b and S1a, S1b, and S2 Figs.
(PDF)

S2 Table Histone mark and bisulfite treated datasets processed, originating from different databases. (PDF)

S3 Table List of all primers applied. Restriction enzyme recognition sites are underlined.
(PDF)

S4 Table Methylation status of CpGs encoded in the 5u *MB* promoter and its potentially interacting DNA regions A1 to G. Bisulfite Sequencing results of each datasets shown in the headline are listed for chromosome 22 genome positions of potential enhancer regions. Due to a lack of sequencing depth information was not provided for all CpG sites. The last column indicates if the DNA site matches a UCSC browser annotated SNP.
(PDF)
