# Supplementary figures and images for: The Distinct Gene Regulatory Network of Myoglobin in Prostate and Breast Cancer
Source: PLoS One. 2015 Nov 11;10(11):e0142662. doi: 10.1371/journal.pone.0142662 (PMC4641586; doi:10.1371/journal.pone.0142662)

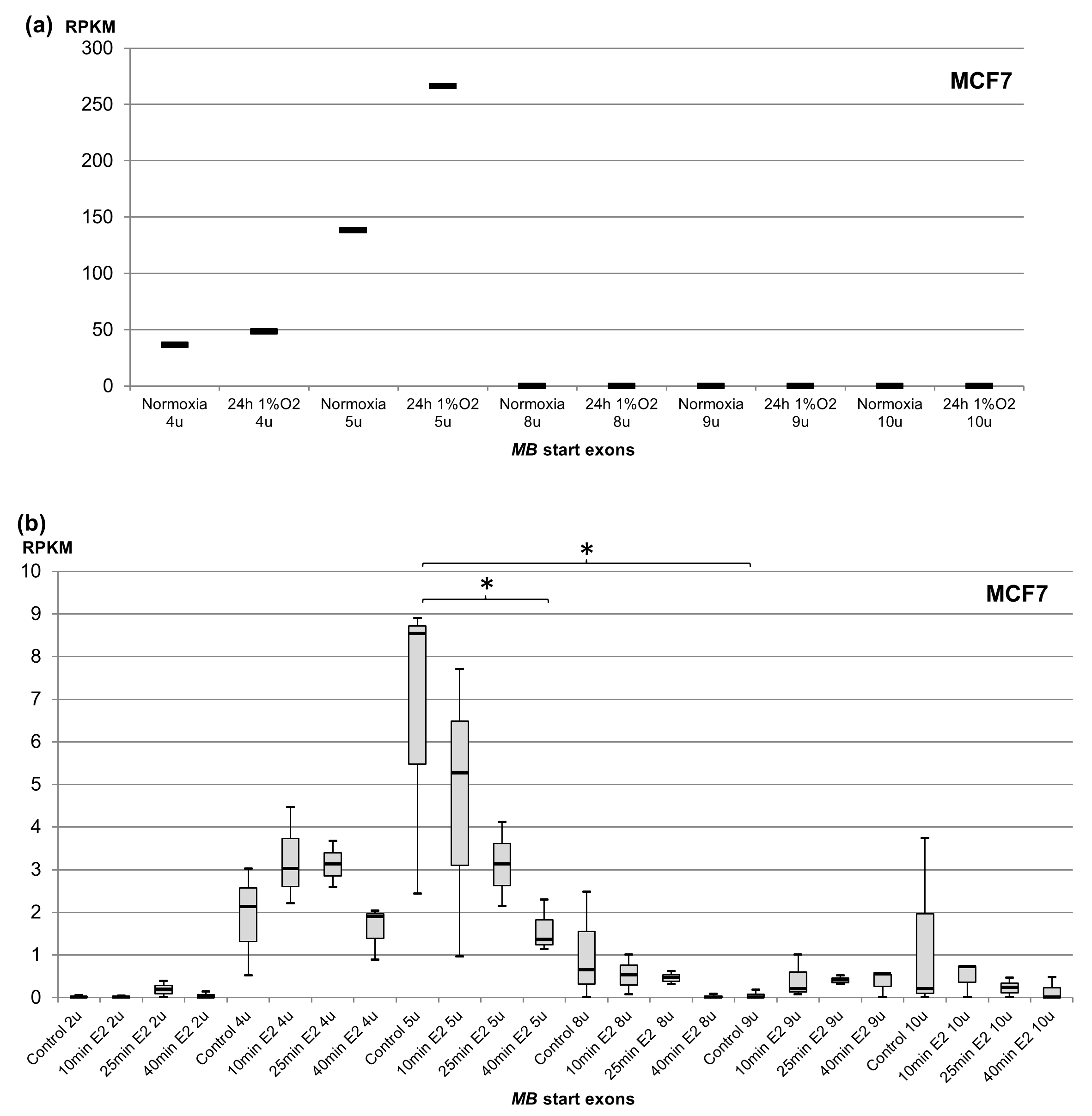

Supplement: S1 Fig — (a) In silico quantification of MB transcripts by RNA-Seq analysis in normoxic and hypoxic (1% O2, 24 h) cancer cells. Read counts are shown as RPKM values. Expression values are detailed in S1 Table. (b) Start-site specific MB expression in MCF7 cells treated with 100 mM E2 for different time periods. GRO-Seq reads which mapped 100 bp upstream to 500 bp downstream to each start site were counted and normalized to the fragments’ size and total reads of the dataset (in Mio). Box plots of (n = 2–3) datasets for each time period represent the transcriptional levels of MB start exons (* p < 0.05). Average transcript-specific expression values are given in S1 Table. (TIF) [file pone.0142662.s001.tif]

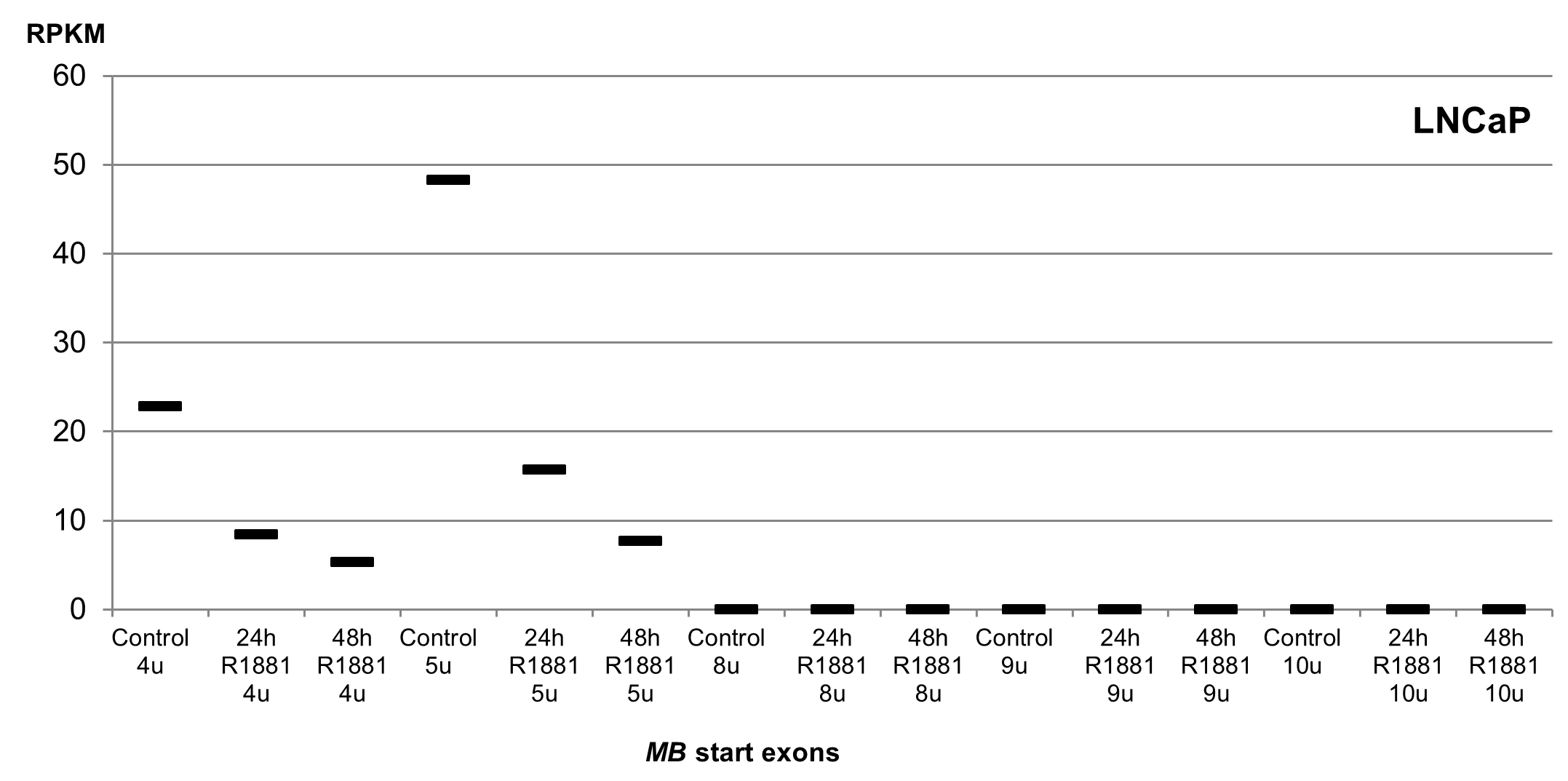

Supplement: S2 Fig — MB start exon quantifications are shown as RPKM values for each experiment, indicating the expression of according mRNA variants. Expression values are detailed in S1 Table. (TIF) [file pone.0142662.s002.tif]

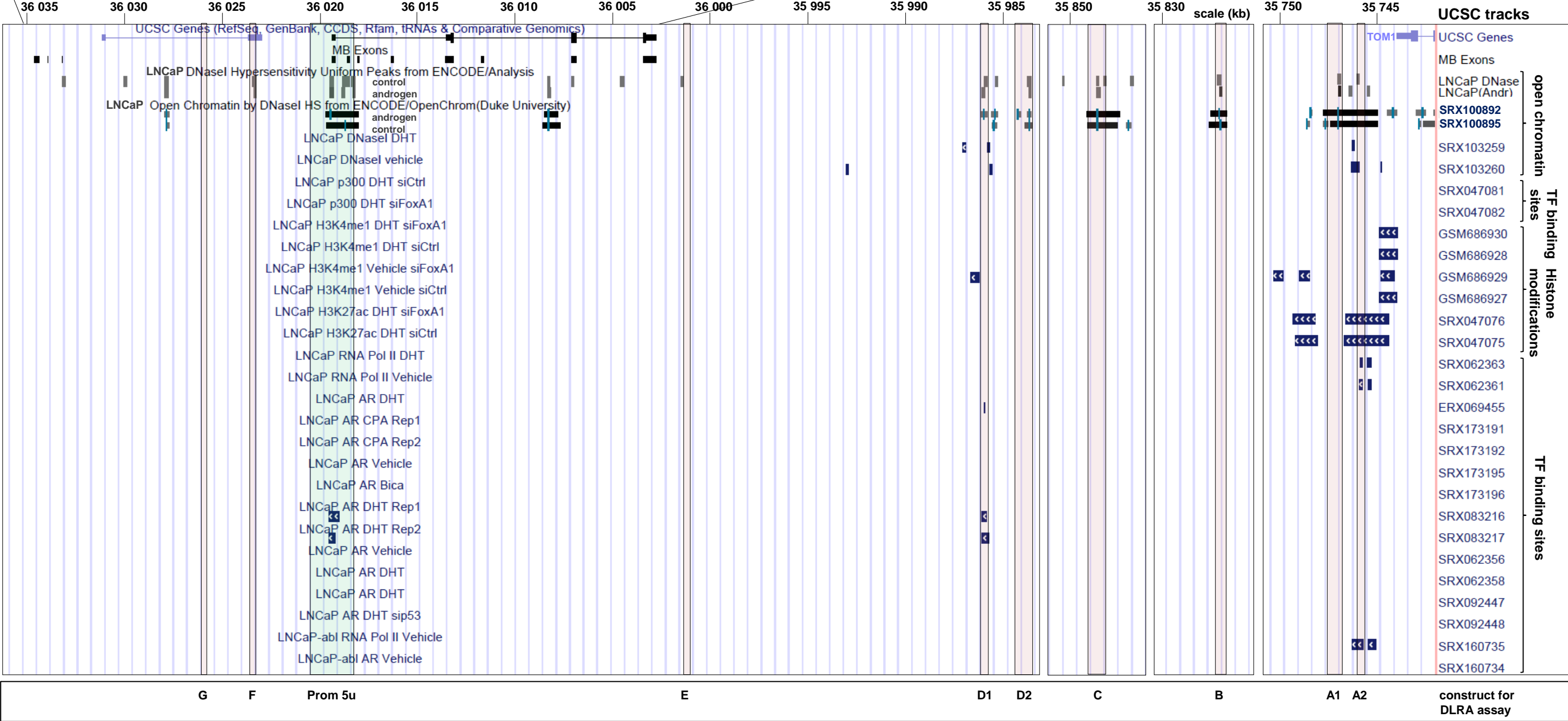

**(b)** RLU fold increase

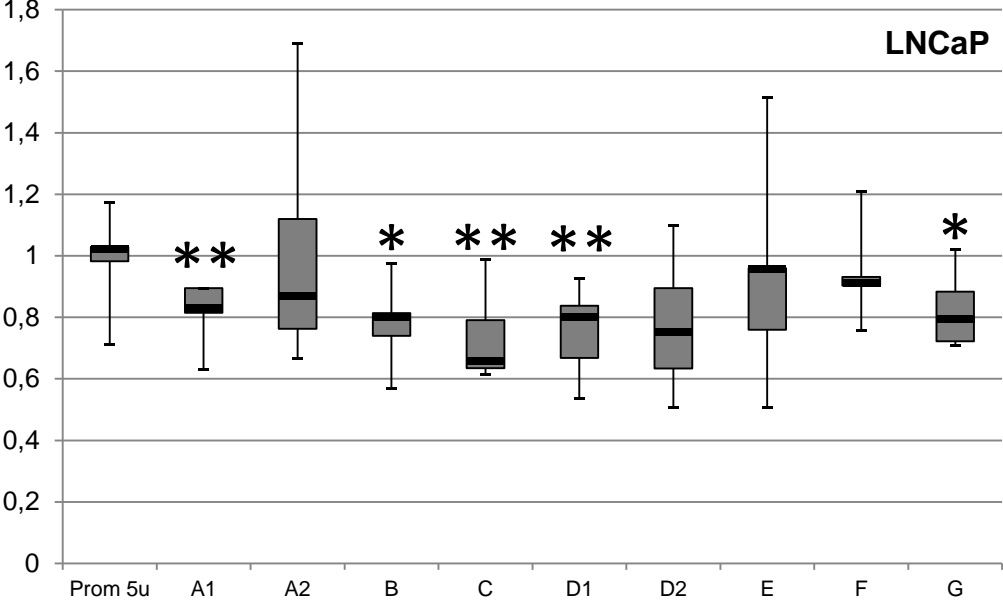

Supplement: S3 Fig — (a) UCSC browser overview of androgen associated chromatin modifications around the 5u MB promoter in LNCaP cells. Top: UCSC annotation of MB and a custom track of all human MB exons, according to [21]. Bottom: UCSC browser and custom tracks. Dataset specifications are listed on the right. The green shattered box indicates the 5u MB promoter region. The red shattered boxes mark the DNA regions that directly interact with the 5u MB promoter based on ChIA-PET data. The naming of the regions to match the DLRA measured constructs is written in the bottom line. (b) Dihydrotestosterone inducibility of the MB 5u promoter and interacting DNA regions in LNCaP cells. DLRAs were measured on 100 nM DHT treated (for 3 h) and control cells transfected with reportergene plasmids with different DNA regions. Box plots indicate the average RLU fold change of each construct measured in hormone treated versus control cells after normalization on empty vector constructs and renilla control vectors. Standard deviations are indicated by error bars (* p < 0.05; ** p < 0.01; n = 5). (PDF) [file pone.0142662.s003.pdf]

(a)

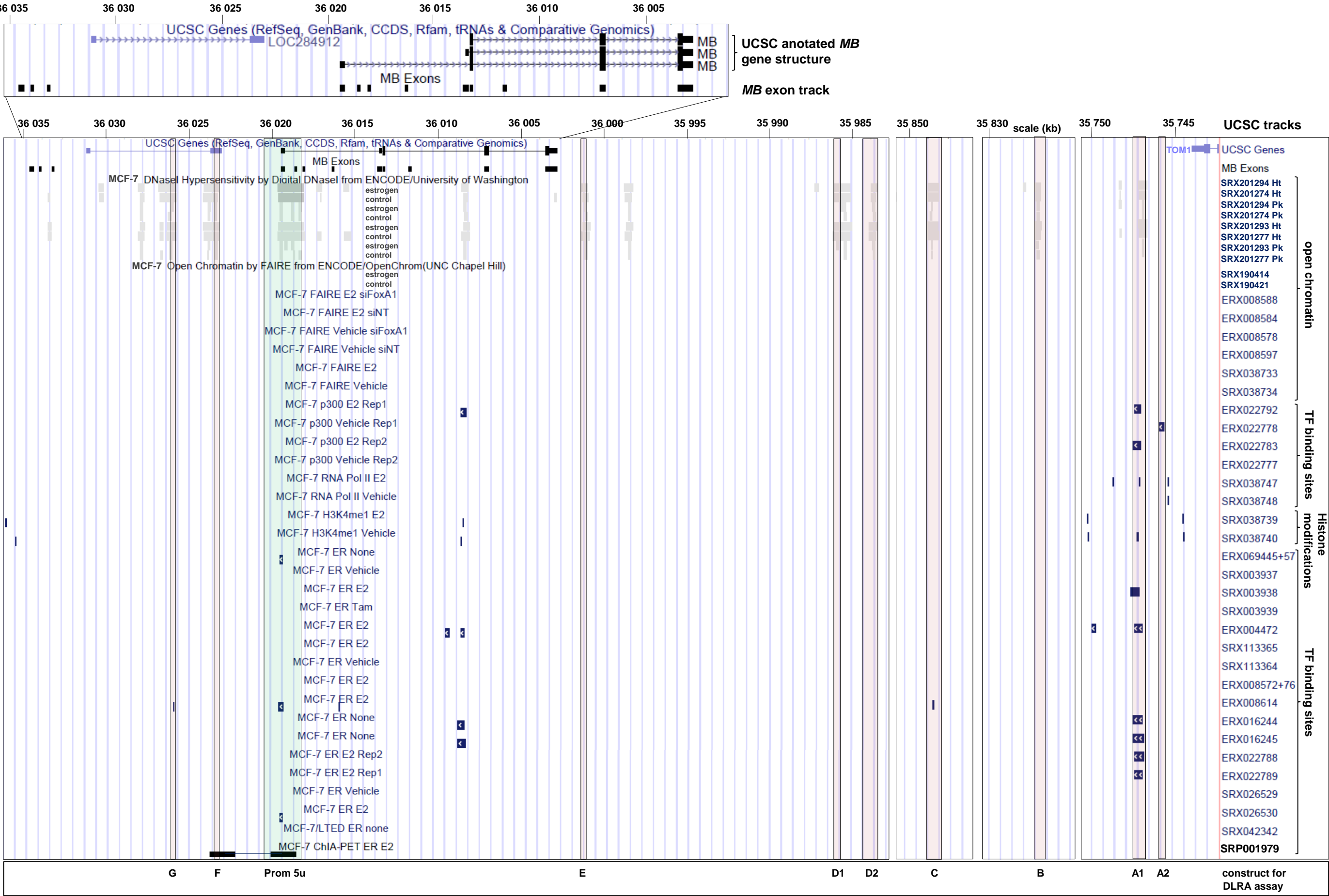

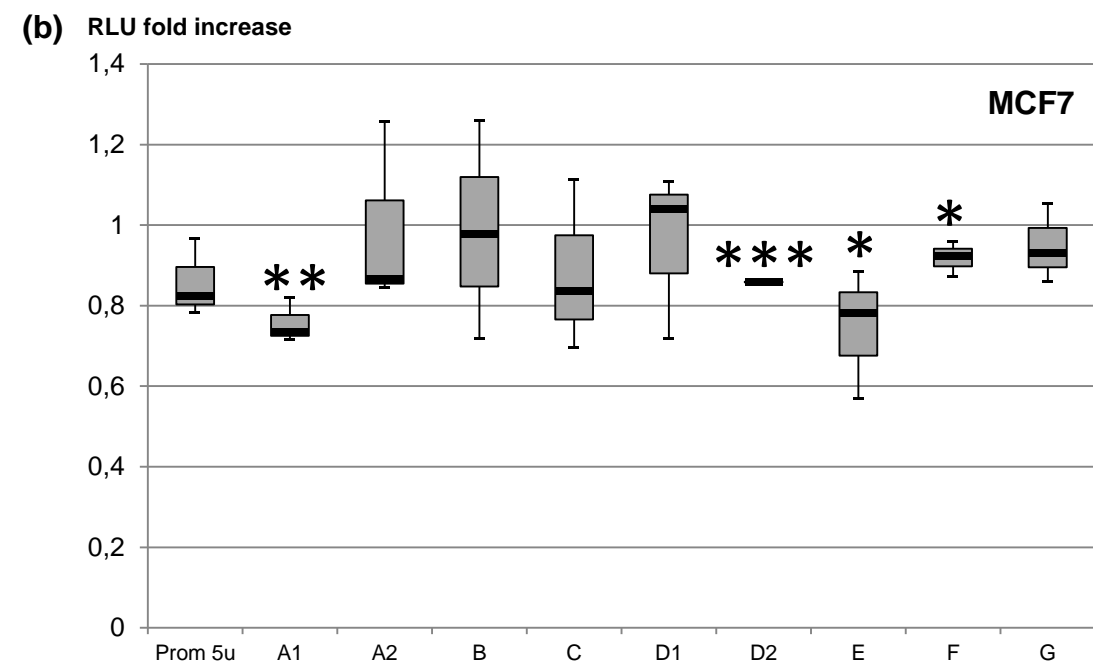

Supplement: S4 Fig — (a) UCSC browser overview of estrogen associated chromatin modifications around the 5u MB promoter in MCF7 cells. Top: UCSC annotation of the human MB gene and a custom track of all human MB exons, according to [21]. Bottom: UCSC browser and custom tracks. Dataset specifications are listed on the right. The green shattered box indicates the 5u MB promoter region. The red shattered boxes mark the DNA regions that directly interact with the 5u MB promoter based on ChIA-PET data. The naming of the regions to match the DLRA measured constructs is written in the bottom line. (b) Estrogen inducibility of the MB 5u promoter and interacting DNA regions in MCF7 cells. DLRAs were conducted on 100 nM E2 treated (for 1 h) and control cells transfected with reportergene plasmids with different DNA regions. Box plots indicate the average RLU fold change of each construct measured in hormone treated versus control cells after normalization on empty vector constructs and renilla control vectors. Standard deviations are indicated by error bars (* p < 0.05; ** p < 0.01; *** p < 0.001; n = 3). (PDF) [file pone.0142662.s004.pdf]
