# Supplementary material for: The Distinct Gene Regulatory Network of Myoglobin in Prostate and Breast Cancer
Source: PLoS One. 2015 Nov 11;10(11):e0142662. doi: 10.1371/journal.pone.0142662 (PMC4641586; doi:10.1371/journal.pone.0142662)
Supplement: S1 Table — RNA-Seq reads mapping to each MB start exon were counted and normalized as RPKM values in order to estimate the average start-site specific expression of different MB variants. Detailed box plots of all studies are shown in Figs 1c, 2a and 2b and S1a, S1b, and S2 Figs. (PDF) [file pone.0142662.s005.pdf]

S1 Table: RNA-Seq and GRO-Seq datasets analyzed from the NCBI-Sequence Read Archive (SRA) with resulting *MB* start exon expression values.

SRA number	Sequencing Technique	Cell line	Treatment	Average RPKM value for *MB* start exon						
				1u	2u	4u	5u	8u	9u	10u
SRR1012917-18	RNA-Seq	MCF7	24h 10nM E2	0	0	1.72	2.93	0	0	0.03
SRR1012919-20										
SRR1012921-22										
SRR1012923-24										
SRR1012925-27										
SRR1012928-30										
SRR1012931-33										
SRR1012934-36			Control	0	0	3.11	7.37	0.02	0	0.11
SRR1012937-39										
SRR1012940-42										
SRR1012943-45										
SRR1012946-48										
SRR1012949-51										
SRR1012952-54										
SRR579319-23	GRO-Seq	MCF7	Control	0	0.02	1.89	6.62	1.04	0.06	1.31
SRR579310-18										
SRR579299-09										
SRR579324-34			10min 100nM E2	0	0.01	3.23	4.64	0.53	0.43	0.49
SRR579335-41										
SRR579342-46										
SRR579347-52			25min 100nM E2	0	0.19	3.13	3.13	0.46	0.41	0.23
SRR579353-59										
SRR579360-63			40min 100nM E2	0	0.05	1.60	1.59	0.03	0.36	0.16
SRR579364-69										
SRR579370-78										
SRR013349, SRR013351	RNA-Seq	MCF7	Normoxia	0	0	36.67	138.25	0	0	0
SRR013350			24h 1% O_2	0	0	48.50	266.08	0	0	0
SRR090594	RNA-Seq	LNCaP	Control	0	0	22.81	48.28	0	0	0
SRR090595			24h 1nM R1881	0	0	8.40	15.67	0	0	0
SRR090596			48h 1nM R1881	0	0	5.27	7.65	0	0	0
SRR393689-90	RNA-Seq	LNCaP	Control	0	0	21.76	25.65	0.04	0.12	0.06
SRR393691-92										
SRR393693										
SRR393694-95			1nM R1881 12h	0	0.13	17.91	16.54	0.04	0	0
SRR393696-97										
SRR393698-99										
ERR365984	RNA-Seq	MCF7	Normoxia	0	0	1.2	1.27	0.08	0	0.25
ERR365993										
ERR365989			24h 1% O_2	0	0	1.45	5.45	0	0	0
ERR365994										
