# Supplementary material for: The Distinct Gene Regulatory Network of Myoglobin in Prostate and Breast Cancer
Source: PLoS One. 2015 Nov 11;10(11):e0142662. doi: 10.1371/journal.pone.0142662 (PMC4641586; doi:10.1371/journal.pone.0142662)
Supplement: S2 Table — (PDF) [file pone.0142662.s006.pdf]

S2 Table: Histone mark and bisulfite treated datasets processed, originating from different databases.

SRA-/GEO-Number	Treatment	Sequencing Technique	Cell line	downloaded file	data processing details
ERX022777	control	MCF7 ChIP-Seq p300	MCF7	.bed file from EMBL-EBI	UCSC custom track using GGA
ERX022778	control	MCF7 ChIP-Seq p300			
ERX022783	100nM E2 for 3h	MCF7 ChIP-Seq p300			
ERX022792	100nM E2 for 3h	MCF7 ChIP-Seq p300			
SRX190256	control	MCF7 ChIP-Seq p300		in UCSC (replicates 1-4)	
SRX038739	10nM E2 for 3h	MCF7 ChIP-Seq H3K4me1		.txt file from GEO	saved as .bed file, UCSC custom track using GGA
SRX038740	control	MCF7 ChIP-Seq H3K4me1			
SRX115153	control	MCF7 ChIP-Seq H3K4me1		.fasta file from NCBI SRA	mapping in GMS, peak calling in GGA, UCSC custom track using GGA
SRX153146	control	MCF7 ChIP-Seq H3K27ac		.gff file from GEO	2 replicates merged, formated to .bed file, UCSC custom track using GGA
SRX152075	control	MCF7 ChIP-Seq H3K27ac		in UCSC	
SRX099867	control	MCF7 ChIP-Seq RNA Pol II		.bed file from GEO	UCSC custom track using GGA
SRX099868	control	MCF7 ChIP-Seq RNA Pol II			
SRX038747	10nM E2 for 3h	MCF7 ChIP-Seq RNA Pol II		.txt file from GEO	saved as .bed file, UCSC custom track using GGA
SRX038748	control	MCF7 ChIP-Seq RNA Pol II			
SRX102994	control	MCF7 ChIP-Seq RNA Pol II		in UCSC	
GSM700944	0.5% O_2 for 16h	MCF7 ChIP-Seq HIF1α		.fasta file from SRA	mapping in GMS, peak calling in GGA, UCSC custom track using GGA
GSM700945	0.5% O_2 for 16h	MCF7 ChIP-Seq HIF2α			
GSM700947	0.5% O_2 for 16h	MCF7 ChIP-Seq HIFβ			
ERX008572+76	E2	MCF7 ChIP-Seq ERα		.fastq file from Array-Express	mapping in GMS, merged with ERX008576, peak calling in GGA, UCSC custom track using GGA
ERX008572+76	E2	MCF7 ChIP-Seq ERα			mapping in GMS, merged with ERX008572, peak calling in GGA, UCSC custom track using GGA
ERX008614	E2	MCF7 ChIP-Seq ERα			mapping in GMS, peak calling in GGA, UCSC custom track using GGA
ERX069445+57	100nM R1881, 1µM bicalutamide	MCF7 ChIP-Seq ERα			mapping in GMS, merged with ERX069445, peak calling in GGA, UCSC custom track using GGA
ERX069445+57	100nM R1881, 1µM bicalutamide	MCF7 ChIP-Seq ERα			mapping in GMS, merged with ERX069457, peak calling in GGA, UCSC custom track using GGA
	control	MCF7 ChIP-Seq input			mapping in GMS, as input control to ERX069445 and ERX069457
SRX113364	control	MCF7 ChIP-Seq input		.fasta file from Array-Express	mapping in GMS, as input control to SRX113365
SRX113365	100nM E2 for 1h	MCF7 ChIP-Seq ERα			mapping in GMS, peak calling in GGA, UCSC custom track using GGA
ERX004472	control	MCF7 ChIP-Seq ERα		.gff files from Array-Express	saved as .bed file in GGA, UCSC custom track using GGA

SRX026529	control	MCF7 ChIP-Seq ERα	MCF7	.fasta file from SRA	mapping in GMS, peak calling in GGA, UCSC custom track using GGA
SRX026530	100nM E2 for 45min	MCF7 ChIP-Seq ERα			
	control	MCF7 ChIP-Seq input			mapping in GMS, as input control to GSM589236 and GSM589237
SRX042342	LTED	MCF7/LTED ChIP-Seq ERα		.txt file from GEO	saved as .bed file in GGA, UCSC custom track using GGA
ERX016244	control	MCF7 ChIP-Seq ERα		.bed file from Array-Express	UCSC custom track using GGA
ERX016245	control	MCF7 ChIP-Seq ERα			
SRX003937	control	MCF7 ChIP-Seq ERα		.bed file from GEO	UCSC custom track using GGA
SRX003938	10nM E2 for 1h	MCF7 ChIP-Seq ERα			
SRX003939	1μM Tamoxifen for 1h	MCF7 ChIP-Seq ERα			
ERX022788	100nM E2 for 3h	MCF7 ChIP-Seq ERα		.bed file from Array-Express	UCSC custom track using GGA
ERX022789	100nM E2 for 3h	MCF7 ChIP-Seq ERα			
SRX100917	10mM Lactate (pH 6.7) 1% O_2 for 24h	MCF7 DNaseIHS-Seq		in UCSC	
SRX189395	control	MCF7 DNaseIHS-Seq		in UCSC	
SRX201294	100nM E2 for 1h	MCF7 DNaseIHS-Seq		in UCSC	
SRX201293	100nM E2 for 1h	MCF7 DNaseIHS-Seq		in UCSC	
SRX201277	control	MCF7 DNaseIHS-Seq		in UCSC	
SRX201274	control	MCF7 DNaseIHS-Seq		in UCSC	
SRX069174	control	MCF7 DNaseIHS-Seq		in UCSC	
SRX069181	control	MCF7 DNaseIHS-Seq		in UCSC	
SRX100886	control	MCF7 DNaseIHS-Seq		in UCSC	
ENCODE UCSC track	control	MCF7 DNaseIHS-Seq		in UCSC	
SRX038733	10nM E2 for 3h	MCF7 FAIRE-Seq		.txt file from GEO	saved as .bed file, UCSC custom track using GGA
SRX038734	control	MCF7 FAIRE-Seq			
SRX190414	10nM E2 for 30min	MCF7 FAIRE-Seq		in UCSC	
SRX117788	10mM Lactate (pH 6.7) 1% O2 for 24h	MCF7 FAIRE-Seq		in UCSC	
SRX190421	control	MCF7 FAIRE-Seq		in UCSC	
ERX008597	control, siNT	MCF7 FAIRE-Seq		.fastq file from Array-Express	mapping in GMS, peak calling in GGA, UCSC custom track using GGA
ERX008578	control, siFoxA1	MCF7 FAIRE-Seq			
ERX008584	E2, siNT	MCF7 FAIRE-Seq			
ERX008588	E2, siFoxA1	MCF7 FAIRE-Seq			
SRR097807	control	MCF7 BS-Seq		.fasta file from SRA	mapping in GMS, filtered for Chr22 by Galaxy, custom track using GGA
SRR201783	control	MCF7 BS-Seq			
SRR201784	control	MCF7 BS-Seq			

SRR201785	control	MCF7 BS-Seq			
SRR222426	control	MCF7 BS-Seq	MCF7	in UCSC	
SRR222420	control	MCF7 BS-Seq		in UCSC	
SRR201782	control	MCF7 BS-Seq		.fasta file from SRA	mapping in GMS, filtered for Chr22 by Galaxy, custom track using GGA
SRX039415	control	MCF7 BS-Seq			
SRX039416	control	MCF7 BS-Seq			
SRX160879	control	MCF7 ChIA-PET RNA Pol II		in UCSC (replicates 1-4)	
SRX160885	control	MCF7 ChIA-PET CTCF		in UCSC (replicates 1-2)	
SRX160882	control	MCF7 ChIA-PET ERα		in UCSC (replicates 1-3)	
SRX107344	control	MCF7 ChIA-PET RNA Pol II		.txt file from GEO	formated to .bed file, UCSC custom track using GGA
SRX107345	control	MCF7 ChIA-PET RNA Pol II			
SRX107346	control	MCF7 ChIA-PET RNA Pol II			
SRX107347	control	MCF7 ChIA-PET RNA Pol II			
SRP001979	100nM E2 for 45min	MCF7 ChIA-PET ERα		genome coordinates from ChIA-PET Browser	generation of genomic coordinate file for SRX016696, SRX016697, SRX016699 and SRX016700, all merged as SRP001979
SRP001979	100nM E2 for 45min	MCF7 ChIA-PET ERα			
SRP001979	100nM E2 for 45min	MCF7 ChIA-PET ERα			
SRP001979	100nM E2 for 45min	MCF7 ChIA-PET ERα			
SRX047081	DHT for 1h, siCTRL	LNCaP ChIP-Seq p300	LNCaP	.bed file from GEO	UCSC custom track using GGA
SRX047082	DHT for 1h, siFoxA1	LNCaP ChIP-Seq p300			
GSM686927	control siCTRL	LNCap ChIP-Seq H3K4me1			
GSM686928	DHT for 1h, siCTRL	LNCap ChIP-Seq H3K4me1			
GSM686929	Control, siFoxA1	LNCap ChIP-Seq H3K4me1			
GSM686930	DHT for 1h, siFoxA1	LNCap ChIP-Seq H3K4me1			
SRX047075	DHT for 1h, siCTRL	LNCaP ChIP-Seq H3K27ac			
SRX047076	DHT for 1h, siFoxA1	LNCaP ChIP-Seq H3K27ac			
SRX062361	control	LNCaP ChIP-Seq RNA Pol II			
SRX062363	100nM DHT for 2h	LNCaP ChIP-Seq RNA Pol II			
SRX160735	control	LNCaP ChIP-Seq RNA Pol II			
SRX062356	control	LNCaP ChIP-Seq AR		.fasta file from SRA	mapping in GMS, peak calling in GGA, UCSC custom track using GGA
SRX062358	100nM DHT for 2h	LNCaP ChIP-Seq AR			
	control	LNCaP ChIP-Seq input		.fasta file from SRA	mapping in GMS, as input control to SRX062356 and SRX062358
	control	LNCaP ChIP-Seq input		.fasta file from EMBL-EBI	mapping in GMS, as input control to ERX069455
ERX069455	100nM R1881	LNCaP ChIP-Seq AR		.fasta file from EMBL-EBI	mapping in GMS, peak calling in GGA, UCSC custom track using GGA
SRX173191	1000nM CPA for 2h	LNCaP ChIP-Seq AR		.bed file from GEO	UCSC custom track using GGA

SRX173192	1000nM CPA for 2h	LNCaP ChIP-Seq AR	LNCaP	.bed file from GEO	UCSC custom track using GGA
SRX173195	control	LNCaP ChIP-Seq AR			
SRX173196	1000nM Bica for 2h	LNCaP ChIP-Seq AR			
SRX083216	100nM DHT for 2h	LNCaP ChIP-Seq AR			
SRX083217	100nM DHT for 2h	LNCaP ChIP-Seq AR			
SRX092447	10nM DHT for 1h	LNCaP ChIP-Seq AR			
SRX092448	10nM DHT for 1h, siP53	LNCaP ChIP-Seq AR			
SRX160734	control	LNCaP ChIP-Seq AR			
SRX103259	10nM DHT for 4h	LNCaP DNaseIHS-Seq		.bed file from GEO	peak calling in GGA, UCSC custom track using GGA
SRX103260	control	LNCaP DNaseIHS-Seq			
SRX100892	1nM R1881 for 12h	LNCaP DNaseIHS-Seq		in UCSC	
SRX100895	control	LNCaP DNaseIHS-Seq		in UCSC	
ENCODE UCSC track	1nM R1881 for 12h	LNCaP DNaseIHS-Seq		in UCSC	
ENCODE UCSC track	control	LNCaP DNaseIHS-Seq		in UCSC	
SRX069158	control	LNCaP DNaseIHS-Seq		in UCSC	
SRX069196	control	LNCaP DNaseIHS-Seq		in UCSC	
SRR222492	control	LNCaP BS-Seq		in UCSC	
SRR222493	control	LNCaP BS-Seq		in UCSC	
SRR222573	1nM R1881 for 12h	LNCaP BS-Seq		in UCSC	
SRR222401	control	LNCaP BS-Seq		in UCSC	
SRR222409	1nM R1881 for 12h	LNCaP BS-Seq		in UCSC	
SRR222552	control	LNCaP BS-Seq		in UCSC	
DRX000086_1	1% O_2	DLD-1 ChIP-Seq RNA Pol II	DLD-1	genome coordinates from DBTSS	generation of genomic coordinate file from DBTSS
DRX000086_2	21% O_2	DLD-1 ChIP-Seq RNA Pol II			
SRR097809	control	MDA-MB468 BS-Seq	MDA-MB468	.fasta file from SRA	mapping in GMS, filtered for Chr22 by Galaxy, custom track using GGA
GSM999338	control	HepG2 BS-Seq	HepG2	in UCSC	
GSM999364	control	HUVEC BS-Seq	HUVEC	in UCSC	
