# Supplementary material for: The Distinct Gene Regulatory Network of Myoglobin in Prostate and Breast Cancer
Source: PLoS One. 2015 Nov 11;10(11):e0142662. doi: 10.1371/journal.pone.0142662 (PMC4641586; doi:10.1371/journal.pone.0142662)
Supplement: S3 Table — Restriction enzyme recognition sites are underlined. (PDF) [file pone.0142662.s007.pdf]

S3 Table: List of all primers applied. Restriction enzyme recognition sites are underlined.

Name	Sequence (5’ --- 3’)	Application
Primer A1 for	NNNNACGCGTCAGGTTTCTCACTGCTGGAG	Generation of reportergene plasmids
Primer A1 rev	NNNNCTCGAGGTAGGCCCAGTTCTGAGTC	
Primer A2 for	NNNNACGCGTGACAGCTGGCCCTCATTTTC	
Primer A2 rev	NNNNCTCGAGAAGATGGGACGGGACGC	
Primer B2 for	GAATACGCGTCGATAACTCAGGCATCCCC	
Primer B2 rev	GAATCTCGAGCCTGACCTCCTGATCCAC	
Primer C for	CGATACGCGTGCAAAGCAGGGATAGCGG	
Primer C rev	CGTACTCGAGGCTTGGGCATCACTTGACC	
Primer D1 for	GAATACGCGTCTGTCCAGGGCTAGAGG	
Primer D1 rev	GTAACTCGAGCAGGCAACATAGTGAGACC	
Primer D2 for	GTAAACGCGTGTACCTGCCTTGCAAGG	
Primer D2 rev	GAATCTCGAGCCACATGGCACTGTCTG	
Primer E for	GAATACGCGTCCCAGTTTATGTGCACC	
Primer E rev	GAATCTCGAGCAATGACTAAGGACCAGG	
Primer F for	GTAAACGCGTGGATGGCTGTGTATCCC	
Primer F rev	GTAACTCGAGTTCGGAGCTGAAGGAGAGG	
Primer G for	NNNNACGCGTAGAGGCATGAAAGGTGGTAAG	
Primer G rev	NNNNCTCGAGCAAGTTCAAAATCATTCCCCATG	
Primer Prom Ex5u for	NNNNACGCGTTACACTGTGCCAATCACCGC	
Primer Prom Ex5u rev	NNNNAAGCTTCAAGCTTGCTCCAGACTCCC	
Primer Ex4u for	GCATGTTGGCCTGGTCCTTTGC	qRT-PCR on Variant 13
Primer Ex9u for	CCCAGTGAGCCCATACTTGC	qRT-PCR on Variant 2
Primer Ex9c/11c rev	CTTAAAGAGCCTGATGAGGAC	qRT-PCR on Variants 2 and 13
Primer Ex5u/9c for	GAGCTGTGACTGCGCCATG	qRT-PCR on Variant 9
Primer Ex6u/9c for	TGTGCAGACTGCGCCATG	qRT-PCR on Variant 10
Primer Ex7u/9c for	GACTACAGACTGCGCCATG	qRT-PCR on Variant 11
Primer Ex11c rev	GCCTTCATCTCGTCCTCTGAC	qRT-PCR on Variants 9,10 and 11
Primer LMNB1 for	GAAGAAGAGAGGTTGAAGCTG	qRT-PCR on LMNB1
Primer LMNB1 rev	CTAACACTACTACTCGCCTCTG	qRT-PCR on LMNB1
