# Supplementary material for: The Distinct Gene Regulatory Network of Myoglobin in Prostate and Breast Cancer
Source: PLoS One. 2015 Nov 11;10(11):e0142662. doi: 10.1371/journal.pone.0142662 (PMC4641586; doi:10.1371/journal.pone.0142662)
Supplement: S4 Table — Bisulfite Sequencing results of each datasets shown in the headline are listed for chromosome 22 genome positions of potential enhancer regions. Due to a lack of sequencing depth information was not provided for all CpG sites. The last column indicates if the DNA site matches a UCSC browser annotated SNP. (PDF) [file pone.0142662.s008.pdf]

S4 Table: Methylation status of CpGs encoded in the 5u *MB* promoter and its potentially interacting DNA regions A1 to G.

potential enhancer region (genome position)	peak no.	position of CpG on Chr22	SRX039415 (MCF-7)	SRX039416 (MCF-7)	SRR201782 (MCF-7)	SRR201783 (MCF-7)	SRR201784 (MCF-7)	SRR201785 (MCF-7)	SRR097807 (MCF-7)	SRR222420 (MCF-7)	SRR222426 (MCF-7)	SRR097809 (MDA-MB468)	SRR222401 (LNCaP, control)	SRR222409 (LNCaP, 12h, 1nM R1881)	SRR222552 (LNCaP, control)	SRR222492 (LNCaP, control)	SRR222493 (LNCaP, control)	SRR222573 (LNCaP, 12h, 1nM R1881)	GSM999338 (HepG2)	GSM999364 (HUVEC)	is known SNP* (y/n)
**G (36,025,889 - 36,026,059)**	G1	36,025,936	-	UW	-	-	-	-	-	-	-	-	-	-	-	-	-	-	-	-	n
**F (36,023,245 - 36,023,532)**	F1	36,023,441	-	-	-	-	-	-	-	-	-	-	U	U	U	> 50 % M	U	-	-	-	y
	F2	36,023,436	-	-	-	-	-	-	-	-	-	-	U	U	U	50 % M	U	-	-	-	n
	F3	36,023,430	-	-	-	-	-	-	-	-	-	-	U	U	< 50 % M	50 % M	U	-	-	-	n
	F4	36,023,400	-	-	-	-	-	-	-	-	-	-	50 % M	> 50 % M	50 % M	M	U	> 50 % M	-	-	n
	F5	36,023,387	-	-	-	-	-	-	-	-	-	-	> 50 % M	M	50 % M	M	< 50 % M	M	-	-	n
**Prom (36,018,449 - 36,020,475)**	Prom1	36,019,698	UC	UC	-	-	-	-	-	-	-	-	-	-	-	-	-	-	-	-	n
	Prom2	36,019,632	UC	UC	-	-	-	-	-	-	-	-	-	-	-	-	-	-	U	U	n
	Prom3	36,019,618	UW	UC	-	-	-	-	-	-	-	-	-	-	-	-	-	-	U	U	n
	Prom4	36,019,497	-	-	-	-	-	-	-	-	-	-	-	-	-	-	-	-	U	U	n
	Prom5	36,019,471	-	-	-	-	-	-	-	-	-	-	-	-	-	-	-	-	U	U	n
	Prom6	36,019,250	-	-	-	-	-	-	-	-	-	-	-	-	-	-	-	-	M	M	n
	Prom7	36,019,187	-	-	-	-	-	-	-	-	-	-	-	-	-	-	-	-	-	-	n
	Prom8	36,018,612	-	-	-	-	-	-	-	-	-	-	-	-	-	-	-	-	U	M	n
**E (36,001,076 - 36,001,371)**	-		-	-	-	-	-	-	-	-	-	-	-	-	-	-	-	-	-	-	-
**D1 (35,985,788 - 35,986,137)**	-		-	-	-	-	-	-	-	-	-	-	-	-	-	-	-	-	-	-	-
**D2 (35,983,531 - 35,984,347)**	-		-	-	-	-	-	-	-	-	-	-	-	-	-	-	-	-	-	-	-
**C (35,848,138 - 35,848,955)**	C1	35,848,439	-	-	UC	-	-	-	-	-	-	-	-	-	-	-	-	-	-	-	n
	C2	35,848,411	-	-	UC	-	-	-	-	-	-	-	-	-	-	-	-	-	-	-	n
	C3	35,848,385	-	-	-	-	-	-	-	-	-	HW	-	-	-	-	-	-	-	-	n
	C4	35,848,374	-	-	-	-	-	-	-	-	-	HW	-	-	-	-	-	-	-	-	n
	C5	35,848,339	-	-	-	-	-	-	-	-	-	HW	-	-	-	-	-	-	-	-	y
**B (35,826,730 - 35,827,235)**	B1	35,827,234	-	-	-	-	HW	-	HW	-	-	-	-	-	-	-	-	-	-	-	n
	B2	35,827,136	-	-	-	-	-	-	-	-	-	UC	-	-	-	-	-	-	-	-	n
	B3	35,827,081	-	-	HC	HC	UC, MC	UC	U	U	U	UW, MC	U	U	U	U	U	U	-	-	n
	B4	35,827,064	-	-	M	UC, MW	UC	HC	U	U	U	M	U	U	U	U	U	U	-	-	n
	B5	35,827,034	-	-	-	UW	HW	-	U	U	U	U	U	U	U	U	U	U	-	-	n
	B6	35,827,024	-	-	-	-	UW, MW	-	-	-	-	-	-	-	-	-	-	-	-	-	y
**A1 (35,746,834 - 35,747,530)**	A1.1	35,747,442	-	-	-	-	-	-	-	-	-	HC	-	-	-	-	-	-	-	-	n
	A1.2	35,747,421	-	-	-	-	-	-	-	-	-	UC	-	-	-	-	-	-	-	-	n
	A1.3	35,747,407	-	-	-	-	-	-	-	-	-	UC	-	-	-	-	-	-	-	-	n
	A1.4	35,747,160	-	-	-	-	-	-	UW	-	-	UW	-	-	-	-	-	-	-	-	n
	A1.5	35,747,140	-	-	-	-	-	-	-	-	-	UW	-	-	-	-	-	-	-	-	n
	A1.6	35,747,084	-	-	-	-	-	-	UC	-	-	-	-	-	-	-	-	-	-	-	n
	A1.7	35,747,078	-	-	-	-	-	-	UC	-	-	-	-	-	-	-	-	-	-	-	n
	A1.8	35,747,062	-	-	-	-	-	-	UW	-	-	UW	-	-	-	-	-	-	-	-	n
	A1.9	35,747,044	-	-	-	-	-	-	UW	-	-	UW	-	-	-	-	-	-	-	-	n
	A1.10	35,747,029	-	-	-	-	-	-	UW	-	-	UW	-	-	-	-	-	-	-	-	n
	A1.11	35,747,018	-	-	-	-	-	-	HW	-	-	UW	-	-	-	-	-	-	-	-	n
	A1.12	35,746,995	-	-	UC	-	-	-	-	-	-	-	-	-	-	-	-	-	-	-	n
	A1.13	35,746,982	-	-	UC	UC	-	MC	U	U	U	-	U	U	U	U	U	U	-	-	n
	A1.14	35,746,961	-	-	UW, MC	UC, MW	UW	UW, MC	U	U	U	U	U	U	U	U	U	U	-	-	n
	A1.15	35,746,938	-	-	HW	UW	MW	MW	UW	U	U	U	U	U	U	U	U	U	-	-	n
	A1.16	35,746,903	-	-	-	-	-	-	UW	-	-	-	-	-	-	-	-	-	-	-	n
	A1.17	35,746,897	-	-	-	-	-	-	UW	-	-	-	-	-	-	-	-	-	-	-	n
**A2 (35,745,635 - 35,745,988)**	-		-	-	-	-	-	-	-	-	-	-	-	-	-	-	-	-	-	-	-

**M**	CpG on Watson strand and Crick strand methylated
**MW**	CpG on Watson strand methylated
**MC**	CpG on Crick strand methylated
**U**	Watson strand and Crick strand unmethylated
**UW**	CpG on Watson strand unmethylated
**UC**	CpG on Crick strand unmethylated
**-**	track does not provide information on the site's methylation status

**U**	0 % of CpGs methylated
**< 50 % M**	less than 50 % of CpGs methylated
**50 % M**	50 % of CpGs methylated
**> 50 % M**	more than 50 % of CpGs methylated
**-**	track does not provide information on the site's methylation status

*based on UCSC Track dbSNP 138, with a cutoff set for SNP to be prevalent in >= 1% of samples (for details, see http://www.ncbi.nlm.nih.gov/pubmed/11125122)
